# Ventral and Intermediate Hippocampus Are Required for Object-in-Place Recognition Memory in Mice

**DOI:** 10.1523/ENEURO.0105-26.2026

**Published:** 2026-06-16

**Authors:** Arely Cruz-Sanchez, Ryan Appings, Kathleen LaDouceur, Mehreen Inayat, Francesca Violi, Maithe Arruda-Carvalho

**Affiliations:** ^1^Departments of Psychology, University of Toronto, Toronto, Ontario M1C1A4, Canada; ^2^Cell and Systems Biology, University of Toronto, Toronto, Ontario M1C1A4, Canada

**Keywords:** chemogenetics, intermediate CA1, medial prefrontal cortex, mice, recognition memory, ventral CA1

## Abstract

Many behaviors that are essential for survival, such as retrieving food, finding shelter, and locating predator cues, rely on forming effective associations between the identity and location of spatial elements. This identity–location association is commonly assessed in rodents using spontaneous object-in-place (OiP) recognition memory tasks. OiP recognition memory deficits are seen in autism spectrum disorder and schizophrenia and are used to detect early onset of Alzheimer's disease. These deficits are replicated in animal models of neurodevelopmental, neurodegenerative, and chromosomal disorders. The ventral hippocampus (HPC) has been implicated in object recognition, but its contribution to OiP memory has not been established. Despite the broad adoption of mouse models in behavioral and systems neuroscience research for their ease of genetic manipulations, the neural correlates of OiP memory in mice remain unknown. Here we used chemogenetics to assess the contribution of the ventral and intermediate CA1 (vCA1 and iCA1, respectively) subregions of the HPC, as well as the medial prefrontal cortex (mPFC) and iCA1-mPFC projections to OiP memory in male and female C57BL/6J mice. We found that two-object OiP requires the activity of the vCA1 and iCA1, but not the mPFC or iCA1-mPFC connections. Our data identify a requirement for two hippocampal subregions in the successful assessment of OiP recognition memory in mice, expanding our understanding of the neural basis of spatial memory processing.

## Significance Statement

Associations between the identity and location of spatial elements (what–where associations) underlie essential behaviors such as finding food, locating shelter, and safely navigating the environment. Deficits in identity–location processing occur in patients with neurodevelopmental and neurodegenerative disorders and are replicated in rodent models using object-in-place (OiP) recognition tasks. While mice have emerged as a widely used animal model to study the biological mechanisms underlying these disorders, nothing is known about the neural substrates of OiP memory in mice. Here we uncover a novel contribution of the ventral and intermediate hippocampal subregions to OiP performance, deepening our understanding of the neural signatures of spatial memory processing.

## Introduction

An animal's ability to survive relies on the optimal navigation of their environment. This includes being able to identify and locate specific environmental cues, e.g., to find food and shelter or to avoid spaces previously visited by a predator. This “identity–location” association is commonly tested in rodents through the object-in-place (OiP) recognition memory task (Poucet et al., 1986; Granon et al., 1996; Dix and Aggleton, 1999; Ameen-Ali et al., 2015), which captures an animal’s ability to recall an association between a previously encountered object and its specific location. OiP recognition memory is commonly tested in rats using either a four-object (Poucet, 1989; Dix and Aggleton, 1999; Ameen-Ali et al., 2015) or two-object (Eacott and Norman, 2004; Langston and Wood, 2010) task design, in which animals need to identify two (or one) objects that swap positions during the test, thereby assessing the “identity–location” or “what–where” dimension of recognition memory (Dix and Aggleton, 1999; Barker et al., 2007, 2017; Barker and Warburton, 2008, 2015; Ameen-Ali et al., 2015). Importantly, object–location association memory deficits are seen in people with schizophrenia (Wood et al., 2002; Burglen et al., 2004; Wout et al., 2006), and OiP memory deficits are reported in rodent models of neurodevelopmental and neurodegenerative disorders (Howland et al., 2012; Connors et al., 2014; Ballendine et al., 2015; Bonardi et al., 2016, 2021; Hall et al., 2016; Kentner et al., 2016), highlighting the importance of understanding the neural mechanisms underlying OiP memory deficits.

The hippocampus (HPC) is a critical structure for memory processing (Squire and Zola-Morgan, 1991; Squire, 1992, 2004; Eichenbaum, 2001, 2004), with known contributions to object recognition memory (Brown and Aggleton, 2001; Barker and Warburton, 2011a; Warburton and Brown, 2015; Barker et al., 2017). Studies show that whole hippocampal excitotoxic lesions (Liu and Bilkey, 2001; Good et al., 2007; Langston et al., 2010b; Barker and Warburton, 2011b, 2020) and dorsal hippocampal pharmacological manipulations (Barker and Warburton, 2015; Mitchnick et al., 2015; Savalli et al., 2015; Scott et al., 2017; Sabec et al., 2018) impair OiP memory in rats. The HPC is a heterogeneous structure varying in its molecular, input/output projection, and behavior profiles across its dorsal, intermediate, and ventral domains (Jinno et al., 1999; Bannerman et al., 2004; Thompson et al., 2008; Bast et al., 2009; Dong et al., 2009; Fanselow and Dong, 2010; Kheirbek et al., 2013; Strange et al., 2014; Cembrowski et al., 2016; Malik et al., 2016; Milior et al., 2016; Barker et al., 2017; Jarzebowski et al., 2022; Biane et al., 2023), but little research has explored HPC septotemporal contributions to OiP memory. The few studies that conducted HPC subregion manipulations identified a role for the dorsal HPC (Lee et al., 2005; Hunsaker et al., 2007; Barker and Warburton, 2015) and intermediate HPC projections to the medial prefrontal cortex (mPFC; Barker et al., 2017) in OiP tasks in rats. Critically, while the ventral CA1 (vCA1) subregion of the HPC is required for processing the “what,” “where,” and “when” components of object recognition tasks (Hunsaker et al., 2008; Beer et al., 2014; Neugebauer et al., 2018; Lin et al., 2021), its involvement in “what–where” tasks such as in OiP has never been tested.

The powerful combination of transgenic and viral strategies in mouse models yields a broad array of flexible systems neuroscience tools that have not yet been applied to the study of the neural basis of OiP recognition memory. This is in line with the paucity of studies assessing OiP memory in mice, many of which combine OiP with other recognition memory measures (such as object location and temporal order recognition) within the same session (Ricceri et al., 2000; Roullet et al., 2001; Calamandrei et al. 2002a,b; Castilla-Ortega et al., 2012; Place et al., 2012; Sobin et al., 2017). We recently confirmed robust OiP memory in C57BL/6J mice using a two-object version of the OiP task (Inayat et al., 2026), but the neural underpinnings of OiP memory in mice have not been explored. Here we used designer receptors exclusively activated by designer drugs (DREADDs) to first test the contribution of the HPC subregion vCA1 to OiP memory in C57BL/6J mice. Given reports implicating intermediate CA1 (iCA1)-mPFC projections (Barker et al., 2017) and the mPFC (Barker and Warburton, 2008, 2009, 2011a, 2015; Savalli et al., 2015; Benn et al., 2016; Barker et al., 2017, 2020; Sabec et al., 2018) in four-object OiP in rats, we also assessed the contribution of iCA1, mPFC, and iCA1-mPFC projections to OiP memory in mice. We found that the vCA1 and iCA1, but not the mPFC or its input from iCA1, are necessary for allocentric two-object OiP recognition memory in mice.

## Materials and Methods

### Animals

Adult [postnatal days (P) 50–80] male and female C57BL/6J (Jackson Laboratory, Strain # 000664) mice were used for all experiments. Mice were bred at the University of Toronto Scarborough and kept on a 12 h light/dark cycle (lights on at 07:00 h) with access to food and water *ad libitum*. All experiments were conducted during the light cycle. Approximately equal numbers of females and males were used for all experiments. All animal procedures were approved by the Animal Care Committee at the University of Toronto.

### Apparatus and objects

All recognition memory tests were conducted in a 30 × 30 × 30 cm white plexiglass square chamber with a magnetic, glossy, removable base. The base consisted of a 30 × 30 cm plain white magnetic surface. Removable 3 × 3 cm squares were placed in the corner of the boxes to allow for accurate and consistent object placement and were removed prior to testing. Two of the four walls in the chamber contained visual cues that consisted of 8.5 × 11 inch laminated white sheets with six large black dots (diameter 6.5 cm) at the north wall and 5 black stripes (17.5 cm × 2 cm) at the south wall, similar to Lesburguères et al., (2017). The chamber was elevated 41 cm off the floor with a camera mounted 75 cm above it with a wall mount rack. An external LED lamp (280 lux) was located at the center of and 75 cm above the four chambers to allow even distribution of light. The objects were designed using SolidWorks and 3D printed using a LulzBot TAZ 6 3D printer with natural PLA or white PETG filament [see Inayat et al. (2021) for freely available object designs]. A round magnet (35 mm diameter) was glued to the base of the objects to allow for stable attachment to the chamber floor. All objects had a pegged-surface with the following dimensions: 46 × 46 × 48 mm (step) and 47 mm diameter × 48 mm height (dome). Object designs were extensively piloted to generate objects that were (1) equal in surface area, (2) made of the same materials, and (3) for which the animals displayed no innate preference (Inayat et al., 2021).

### Experimental design and statistical analysis

#### Behavioral testing

##### Handling and habituation

Mice were handled and habituated to the behavioral chamber twice a day for 4 consecutive days prior to the day of testing for all experiments (Cruz-Sanchez et al., 2020). Handling took place in the testing room with a minimum 3 h interval between handling sessions. Handling and habituation consisted of 5 min of handling followed by placement into the behavioral chamber for 4 min. A 4 × 4 cm weigh boat with 1–2 g of rodent chow was placed at the center of the behavioral chamber during habituation to allow for better acclimation to the chamber. For identification purposes, mice were ear notched during surgery or were tail marked prior to handling.

##### Two-object OiP task

To avoid confounds of repeated testing, dedicated cohorts of mice were used per experiment to ensure that each animal was only tested once. Behavioral chambers and objects were cleaned with water between phases and subjects, and with 70% ethanol at the end of the day. Objects were placed in the northeast and northwest corners of the behavioral chamber, 3 cm away from each wall. Displaced objects were counterbalanced for object type (i.e., dome vs step) and side (i.e., novelty on the right vs on the left).

**Figure 1.**
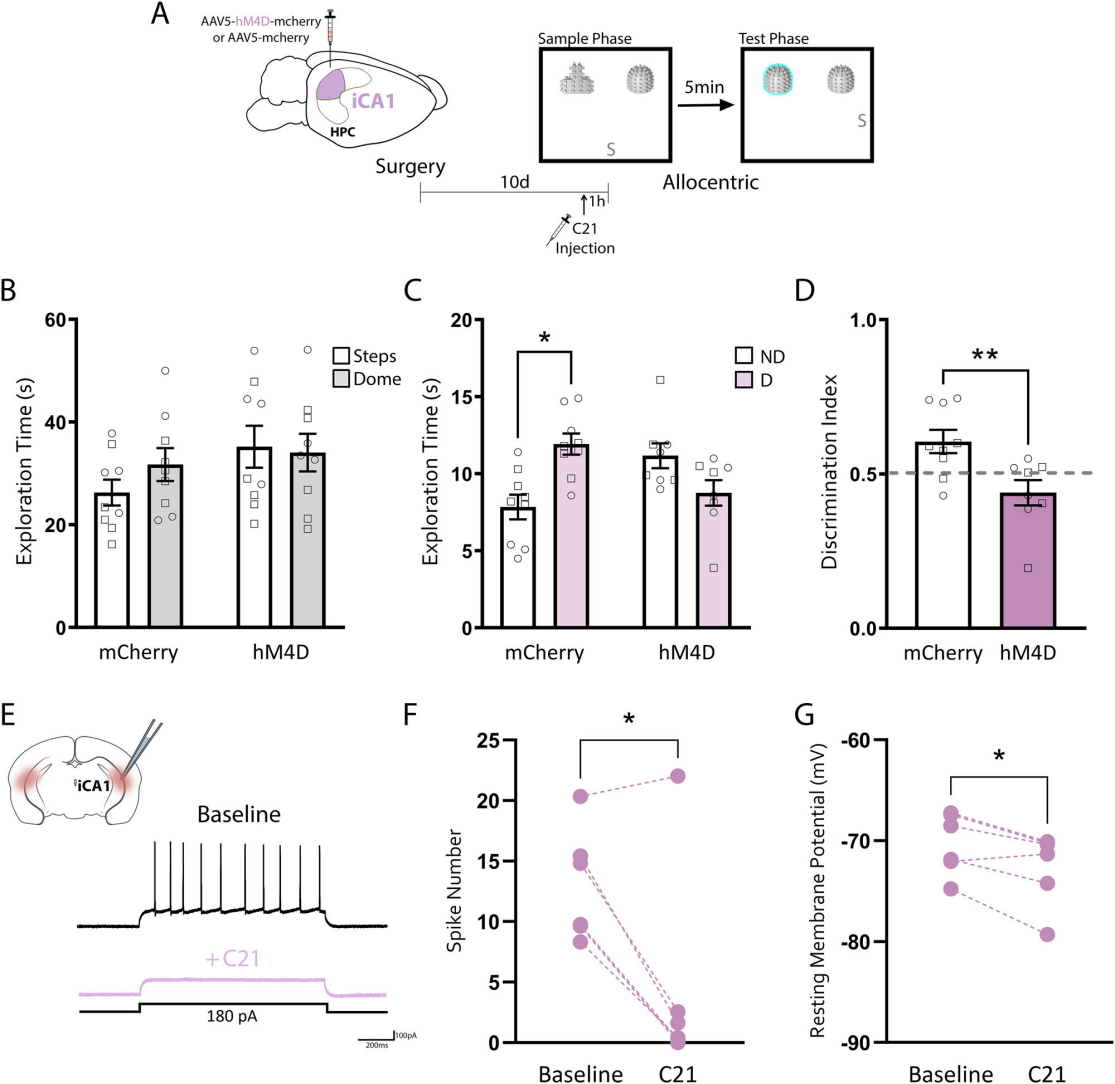
vCA1 activity is required for two-object OiP memory. ***A–D***, vCA1 inhibition during allocentric two-object OiP. ***A***, Experimental timeline. C57BL/6J mice were infused with AAV5-hM4D-mCherry or control AAV5-mCherry into the ventral CA1 (vCA1) subdivision of the hippocampus (HPC). Ten days later, mice were injected with the DREADD agonist C21 1 h prior to testing in the allocentric version of the two-object OiP task (S depicts starting place of animal in chamber). ***B***, mCherry and hM4D vCA1 mice showed no object preference during the sample phase (two-way ANOVA, no treatment × object type interaction; *F*_(1,18)_ = 0.298, *p* = 0.592; or main effect of object: *F*_(1,18)_ = 0.199, *p* = 0.6611 or treatment: *F*_(1,18)_ = 2.610, *p* = 0.1236). ***C***, During the test phase, mCherry vCA1 mice spent more time exploring the displaced (***D***) compared with the nondisplaced (ND) objects, but hM4D vCA1 mice displayed equal preference for both objects. ***D***, A DI (calculated by dividing the amount of time spent exploring the displaced object by the total time spent exploring both the displaced and nondisplaced objects) for each animal showed that hM4D vCA1 animals did not display a preference for the displaced object. mCherry: *n* = 10 (5 females, 5 males); hM4D: *n* = 10 (5 females, 5 males). Individual datapoints from female mice are depicted as circles and male mice as squares for transparency, but no sex differences were found (see Materials and Methods for detailed sex differences statistical analysis). ***E–G***, Electrophysiological validation of vCA1 DREADD experiments. ***E***, Representative trace of action potentials recorded from a vCA1 hM4D-mCherry+ neuron at baseline and after application of C21. ***F***, The number of action potentials evoked by somatic current injection (100 pA) in vCA1 hM4D+ transfected neurons was significantly decreased following bath application of C21 (*n* = 5 cells, 5 mice). ***G***, The resting membrane potential (RMP) of vCA1 hM4D+ neurons became significantly more hyperpolarized following bath application of C21 (*n* = 5 cells, 5 mice). *p < 0.05, **p < 0.001. Extended Data Figure 1-1 shows vCA1 viral target maps.

The OiP memory task comprised one sample phase followed by a test phase (Fig. 1*A*). In the 5 min sample phase (Davis et al., 2013), mice interacted with two objects (dome and step), after which animals were removed and placed back into their home cage. Object identity and location combinations were counterbalanced across animals. After a 5 min delay (Davis et al., 2013; Bonardi et al., 2021), animals underwent a 3 min test phase (Davis et al., 2013) in which they were placed in the chamber with two copies of the same object (two domes or two steps). Given that in the test phase the two objects are familiar and identical, the test measures novelty via identification of the novel object–location contingency (i.e., the copy of the dome object that is positioned where the step object had been, or vice versa). Object type and novelty side (right vs left) at the test phase were also counterbalanced. This task was run in two versions: an egocentric or an allocentric version (Langston and Wood, 2010; Davis et al., 2013), which varied in the placement of the mouse at the start of the test phase to foster different spatial exploration strategies. All experiments were run in the allocentric version except for the iCA1 inhibition in Extended Data Figure 2-2, a control experiment used to confirm that the HPC is not required for egocentric two-object OiP (Langston and Wood, 2010). In egocentric experiments, animals were placed in the chamber with their head facing the wall located opposite the objects (i.e., south wall) for both the sample and test phases (Langston and Wood, 2010; Ainge et al., 2012; Davis et al., 2013). In experiments that used an allocentric paradigm, animals were placed in the chamber with their head facing the south wall during the sample phase and facing either the east or west wall (counterbalanced) during the test phase (Langston and Wood, 2010; Davis et al., 2013).

##### Behavioral analysis

Behavior was analyzed using ANY-maze software (Stoelting). Exploratory activity was defined as in Leger et al. (2013); Cruz-Sanchez et al. (2020). Briefly, object exploration was defined as an object-directed gaze while actively sniffing and/or pawing within 2 cm of the object. Sitting on top of the object while sniffing the surrounding air or chewing the object were not considered exploration. Following Leger et al.’s (2013) recommendation (adapted from Arqué et al., 2008), the analysis of object exploration in the test phase of all experiments comprised only the first 20 s of total interaction time with the objects (Cruz-Sanchez et al., 2020). This is further supported by studies showing rodents demonstrate a higher preference for the novelty within the first 60 to 120 s of the test phase (Dix and Aggleton, 1999; Mumby, 2001; Mumby et al., 2002; Cruz-Sanchez et al., 2020), which corresponds to when mice reached the 20 s criterion in our sample. A discrimination index (DI) was calculated as a measure of relative novelty preference by dividing the amount of time spent exploring the displaced object by the total time spent exploring both the displaced and nondisplaced objects.

### Stereotaxic surgery and viral injections

Mice were anesthetized with isoflurane, and mounted onto a rodent stereotaxic apparatus (Stoelting; Arruda-Carvalho et al., 2017; Botterill et al., 2021). Approximately 0.2 µl of pAAV5-hSyn-hM4D(Gi)-mCherry (≥7 × 10^12^ vg/ml, Addgene # 50475), pAAV5-hSyn-mCherry (≥7 × 10^12^ vg/mL, Addgene # 114472), or AAVrg-hSyn-Cre (≥1.8 × 10^13^ vg/ml, Addgene # 105553) virus was bilaterally infused onto the vCA1 (AP-2.53, ML ±3.25, DV −4.5), iCA1 (AP −2.75, ML ±3.6, DV −3.00), or mPFC (spanning the infralimbic and prelimbic cortices; AP +2.08, ML ±0.26, DV −2.7) using a 10 µl Neuros Syringe (Hamilton Company) and motorized injector (Stoelting; Arruda-Carvalho and Clem, 2014; Arruda-Carvalho et al., 2017). Mice underwent stereotaxic surgery between P50 and P70, with behavioral testing starting 10 d after surgery to allow for recovery. All mice that underwent surgery were given an intraperitoneal (i.p.) injection of the DREADD agonist compound 21 (C21, Hello Bio #HB6124; 2 mg/kg) 1 h prior to the start of OiP testing.

The iCA1 region (pyramidal, oriens, and radiatum layers) was defined as encompassing both Dong et al. (2009)’s posterior dCA1 and iCA1 given their similarity in gene expression (Dong et al., 2009). The region spans from bregmas −2.70 to −3.88 mm in the Franklin & Paxinos mouse atlas (Franklin and Paxinos, 2007). The dorsoventral iCA1 border is set as the lower border of the intermediate CA1 described by Dong et al. (2009) and Fanselow and Dong (2010), which is set above the rhinal fissure/bottom limit of the perirhinal cortex (Franklin and Paxinos, 2007). We followed the vCA1 boundaries set by Dong et al. (2009) and (Fanselow and Dong, 2010). The vCA1 (pyramidal, oriens, and radiatum layers) spans from bregmas −2.92 to −3.88 mm as labeled in the Franklin & Paxinos mouse atlas (Franklin and Paxinos, 2007). The dorsoventral border of vCA1 was set below the rhinal fissure/bottom limit of the perirhinal cortex (Franklin and Paxinos, 2007).

### Slice electrophysiology

We used slice electrophysiology to confirm the effect of C21 on hM4D-transfected neurons in the iCA1, vCA1, or mPFC of adult C57BL/6J mice. Recordings took place following behavioral testing, with a minimum interval of 3 d. Due to resource availability constraints, some animals were delayed by ∼6 months but we did not see an effect of delay on C21 inhibition. Animals were anesthetized with isoflurane and perfused transcardially with ice-cold (0–4°C) sucrose solution composed of the following (in mM): 197 sucrose, 11 glucose, 2.5 KCl, 1.25 NaH_2_PO_4_, 25 NaHCO_3_, 0.4 ascorbate, 1 CaCl_2_, 4 MgCl_2_, and 2 Na^+^ pyruvate. Acute 350 mm coronal slices of iCA1, vCA1, or mPFC were obtained using a VT1000S Vibratome (Leica) and then incubated at 35°C for 25 min in the same solution, but with 50% standard artificial cerebral spinal fluid (ACSF) composed of the following (in mM): 120 NaCl, 2.5 KCl, 1.25 NaH_2_PO_4_, 25 NaHCO_3_, 11 glucose, 2 CaCl_2_, 1 MgCl_2_, and 2 Na^+^ pyruvate. Following recovery, slices were maintained at room temperature in standard ACSF.

Whole-cell current-clamp recordings were obtained from mCherry+ pyramidal neurons in layer 5 of the mPFC (prelimbic and infralimbic cortices) or mCherry+ pyramidal cells in the pyramidal layer of the iCA1 or vCA1 using borosilicate glass electrodes (3–5 MΩ) using an Olympus BX51WI microscope (Evident). Electrode internal solution was composed of the following (in mM): 127.5 K d-gluconate, 10 HEPES, 0.6 EGTA, 5 KCl, 2 MgCl_2_, 2 Mg-ATP, 0.3 Na_2_-GTP, 5 Na-phosphocreatine, and 0.4 Na-GTP. Data were low-pass filtered at 10 kHz and acquired at 10 kHz using MultiClamp 700B and pClamp 10 (Molecular Devices). Series and membrane resistance was continuously monitored, and recordings were discarded when these measurements changed by >20%. Baseline data was acquired by measuring resting membrane potential (RMP) and the number of action potentials evoked by a long (1 s) depolarizing current pulse. Somatic current pulses were calibrated in the baseline period to trigger a minimum of five action potentials in hM4D-mCherry+ pyramidal neurons. After 10 min of stable neuronal discharge induced by a somatic depolarization, C21 (10 µM, HelloBio; Jendryka et al., 2019) was bath applied for at least 10 min following which an additional recording of 10 min was compared with the baseline period.

### Statistical analysis

Data are presented as mean ± SEM. All statistical analyses were performed in GraphPad Prism version 9. Significant statistical results are reported in the main text and nonsignificant results in the figure legends. Exploration time in the sample and test phases was analyzed using two-way, repeated-measures ANOVA followed by Sidak's post hoc tests. A DI comparison between treatment groups was made using an unpaired *t* test. Within each treatment group, one-sample *t* tests comparing DI to chance exploration level of 0.5 was calculated as described previously (Mumby, 2001; Gaskin et al., 2003; Bevins and Besheer, 2006). Object and side preference during the sample and test phases were assessed using a two-way (repeated-measures when necessary) ANOVA followed by Sidak's post hoc tests. For electrophysiology experiments, paired *t* tests were performed to compare C21-mediated cell activity to baseline activity. Potential sex differences were first assessed using a two-way repeated-measures ANOVA, and in the absence of effects, data were collapsed for subsequent analyses.

#### Detailed statistical analyses of potential side, object bias, and sex differences

We did not find innate side preferences (i.e., left vs right object, regardless of object type) during the sample phase [two-way ANOVA treatment × sample phase (left vs right exploration) interaction; vCA1 OiP inhibition: *F*_(1,18)_ = 2.51, *p* = 0.13; iCA1 allocentric OiP inhibition: *F*_(1,19)_ = 0.98, *p* = 0.34; mPFC allocentric OiP inhibition: *F*_(1,22)_ = 0.58, *p* = 0.46; iCA1 egocentric OiP inhibition: *F*_(1,24)_ = 0.069, *p* = 0.80; iCA1-mPFC allocentric OiP inhibition: *F*_(1,28)_ = 0.25, *p* = 0.62]. During the test phase, we did not observe displaced side biases (i.e., preference for whether the displaced object was on the right vs left) in any of the experiments [two-way ANOVA treatment DI × displaced side interaction; vCA1 OiP inhibition: *F*_(1,16)_ = 0.32, *p* = 0.58; iCA1 allocentric OiP inhibition: *F*_(1,17)_ = 3.29, *p* = 0.087; mPFC allocentric OiP inhibition: *F*_(1,20)_ = 2.21, *p* = 0.15; iCA1 egocentric OiP inhibition: *F*_(1,22)_ = 1.51, *p* = 0.23; iCA1-mPFC allocentric OiP inhibition: *F*_(1,26)_ = 1.05, *p* = 0.31]. For allocentric experiments, we found no bias toward chamber placement (facing east or west wall) at the start of the test phase (two-way ANOVA treatment DI × chamber placement interaction; vCA1 OiP inhibition: *F*_(1,16)_ = 0.56, *p* = 0.47; iCA1 allocentric OiP inhibition: *F*_(1,17)_ = 4.01, *p* = 0.061; mPFC allocentric OiP inhibition: *F*_(1,20)_ = 0.061, *p* = 0.11; iCA1-mPFC allocentric OiP inhibition: *F*_(1,26)_ = 0.13, *p* = 0.72).

In addition to the sample phase object bias (e.g., dome vs steps) analyses featured in the results section, we also confirmed an absence of object type bias during the test phase across all experiments (two-way ANOVA treatment DI × object type interaction; vCA1 OiP inhibition: *F*_(1,16)_ = 0.020, *p* = 0.89; iCA1 allocentric OiP inhibition: *F*_(1,17)_ = 0.041, *p* = 0.84; mPFC allocentric OiP inhibition: *F*_(1,20)_ = 0.24, *p* = 0.63; iCA1 egocentric OiP inhibition: *F*_(1,22)_ = 1.24, *p* = 0.28; iCA1-mPFC allocentric OiP inhibition: *F*_(1,26)_ = 2.78, *p* = 0.11). We found no sex differences in any of our behavioral experiments (two-way ANOVA treatment DI × sex interaction: vCA1 OiP inhibition: *F*_(1,16)_ = 3.318, *p* = 0.0873; iCA1 OiP allocentric inhibition: *F*_(1,13)_ = 0.110, *p* = 0.745; iCA1 OiP egocentric inhibition: *F*_(1,22)_ = 0.432, *p* = 0.518; mPFC OiP inhibition: *F*_(1,20)_ = 0.847, *p* = 0.368; iCA1-mPFC OiP inhibition: *F*_(1,26)_ = 0.8498, *p* = 0.365).

## Results

To assess OiP memory in mice, we used a two-object OiP design previously used in rats and mice (Eacott and Norman, 2004; Langston and Wood, 2010; Ainge and Langston, 2012; Davis et al., 2013; Bonardi et al., 2021; Inayat et al., 2026). In this task, mice are initially exposed to two different objects (dome and step) during a sample phase (Fig. 1*A*). Following 5 min, they undergo a test phase in which one of the objects is replaced with an identical copy of the remaining, familiar object (two steps or two domes; Fig. 1*A*). Given that the two objects are identical during the test phase, OiP memory is interpreted as a preference for the replaced (referred to as displaced for consistency within the literature) object, i.e., the object whose identity–location association differs from the one present during the sample phase. As Langston and Wood (2010) showed that the HPC is not required for the two-object OiP task when run in an egocentric configuration, we used an allocentric version of the OiP task. To explore the neural correlates of two-object OiP memory in mice, we used chemogenetics to inhibit the hippocampal subregions vCA1 and iCA1, mPFC, and the iCA1-mPFC projection during OiP.

10.1523/ENEURO.0105-26.2026.f1-1 Figure 1-1 **Viral expression maps of vCA1 manipulations**. Diagrams depicting overlaid viral spread for all vCA1 hM4D infused animals. Download Figure 1-1, TIF file.

### Chemogenetic inhibition of vCA1 impairs two-object OiP memory

Mice were first injected with AAV5-hM4D-mCherry or control AAV5-mCherry into the vCA1 (see Extended Data Fig. 1-1 for viral targeting maps) and underwent the allocentric version of the two-object OiP task (Fig. 1*A*; 5 min retention interval). In this task design, the animals are placed in the chamber facing the wall opposite to the objects (i.e., south wall) in the sample phase and placed facing the west or east wall of the arena in the test phase, precluding the use of an egocentric strategy to distinguish between the object placements (Langston and Wood, 2010; Davis et al., 2013). Mice were injected with the DREADD agonist C21 1 h prior to the start of behavior (Fig. 1*A*). We saw no object bias in the sample phase for either vCA1 mCherry or hM4D mice (Fig. 1*B*). During the test phase, vCA1 hM4D animals showed a deficit in OiP memory (Fig. 1*C*; two-way ANOVA, significant treatment × (nondisplaced vs displaced) object interaction: *F*_(1,18)_ = 7.514, *p* = 0.0134; main effect of object: *F*_(1,18)_ = 7.108, *p* = 0.0157; no effect of treatment: *F*_(1,18)_ = 1.537, *p* = 0.231; Sidak’s post hoc test; mCherry: *t*_(18)_ = 3.824, *p* = 0.0025, hM4D: *t*_(18)_ = 0.0530, *p* = 0.998; Fig. 1*D*; unpaired *t* test: *t*_(18)_ = 7.729, *p* = 0.0138). A within group comparison demonstrated that only the mCherry group obtained a DI significantly greater than chance level of 0.5 (one-sample *t* test; mCherry: *t*_(9)_ = 5.4225, *p* = 0.0005; hM4D: *t*_(9)_ = 0.117, *p* = 0.909). vCA1 mCherry and hM4D animals displayed equivalent total exploration time (total time spent exploring the displaced and nondisplaced objects across the 3 min session; unpaired *t* test: *t*_(18)_ = 0.1198, *p* = 0.906) and distance traveled (unpaired *t* test: *t*_(18)_ = 1.391, *p* = 0.181) during the test phase. Together, these data show that vCA1 activity is necessary for successful OiP recognition memory in the two-object, allocentric version of the OiP task in mice.

We validated our chemogenetic vCA1 manipulation using whole-cell patch-clamp recordings from hM4D+ neurons in the pyramidal layer of the vCA1 (Fig. 1*E–G*). Bath application of C21 resulted in a decrease in spike number (Fig. 1*F*; paired *t* test: *t*_(4)_ = 4.928, *p* = 0.0079) and hyperpolarization of the RMP (Fig. 1*G*; paired *t* test: *t*_(4)_ = 2.961, *p* = 0.0415) in vCA1 hM4D+ pyramidal neurons. These results confirm that acute application of C21 to hM4D-expressing vCA1 pyramidal neurons decreases their excitability.

### Chemogenetic inhibition of iCA1 impairs two-object OiP memory

**Figure 2.**
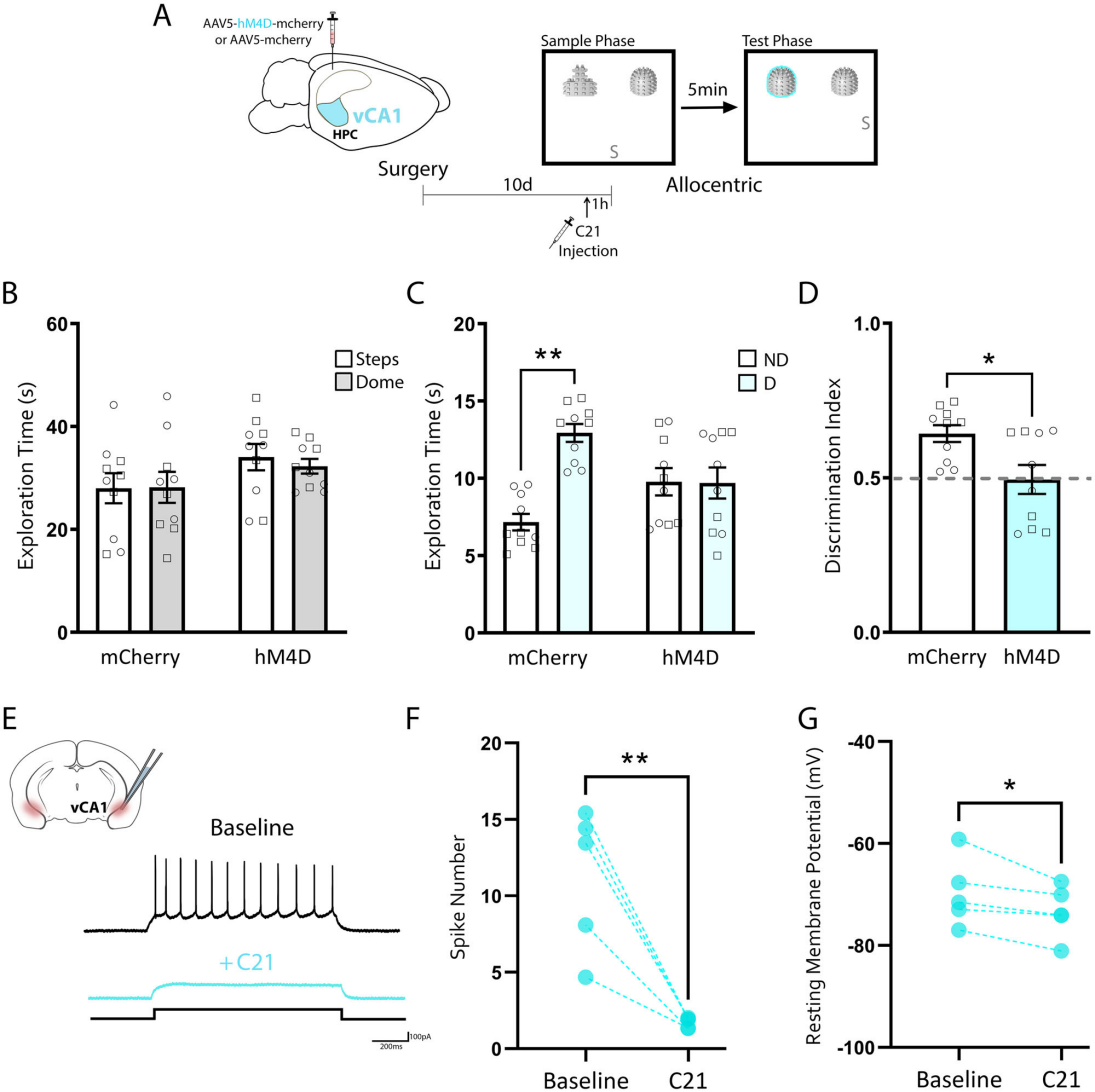
iCA1 activity is necessary for successful two-object OiP memory. ***A–D***, iCA1 manipulation during allocentric two-object OiP. ***A***, Experimental timeline. C57BL/6J mice were infused with AAV5-hM4D-mCherry or control AAV5-mCherry into the intermediate CA1 (iCA1) subdivision of the hippocampus (HPC). Ten days later, mice were injected with the DREADD agonist C21 1 h prior to testing in the allocentric version of the two-object OiP task (S depicts starting place of animal in chamber). ***B***, mCherry and hM4D iCA1 mice showed no object preference during the sample phase (two-way ANOVA, no treatment × object type interaction; *F*_(1,16)_ = 2.055, *p* = 0.1710; main effect of object: *F*_(1,16)_ = 0.8842, *p* = 0.3610 or of treatment: *F*_(1,16)_ = 1.762, *p* = 0.2030). ***C***, During the test phase, mCherry iCA1 mice spent more time exploring the displaced (***D***) compared with the nondisplaced (ND) objects, but hM4D mice displayed equal preference for both objects. ***D***, Calculation of a DI for each animal showed that hM4D iCA1 animals did not display a preference for the displaced object. mCherry: *n* = 9 (4 females, 5 males); hM4D: *n* = 9 (5 females, 4 males). Individual datapoints from female mice are depicted as circles and male mice as squares for transparency, but no sex differences were found. ***E–G***, Electrophysiological validation of iCA1 DREADD experiments. ***E***, Representative trace of action potentials recorded from an iCA1 hM4D-mCherry+ neuron at baseline and after application of C21. ***F***, The number of action potentials evoked by somatic current injection (100 pA) in iCA1 hM4D+ transfected neurons was significantly decreased following bath application of C21 (*n* = 6 cells, 4 mice). ***G***, The resting membrane potential (RMP) of iCA1 hM4D+ neurons became significantly more hyperpolarized following bath application of C21 (*n* = 6 cells, 4 mice). *p < 0.05, **p < 0.01. Extended Data Figure 2-1 shows iCA1 viral target maps and Extended Data Figure 2-2 features iCA1 inhibition during egocentric two-object OiP.

Given the contribution of iCA1-mPFC projections to four-object OiP memory in rats (Barker et al., 2017), we next wanted to test whether iCA1 inhibition would impact OiP memory in mice. Interestingly, our group recently uncovered a differential contribution of the iCA1-mPFC and vCA1-mPFC pathways to cognitive flexibility following developmental disruption (Cruz-Sanchez et al., 2026), suggesting potentially distinct roles for iCA1 and vCA1 subregions in cognitive processing. To test that possibility, we used an identical strategy as the previous experiment, but injected AAV5-hM4D-mCherry or control AAV5-mCherry into the iCA1 (Fig. 2*A*; see Extended Data Fig. 2-1 for viral targeting maps). Neither iCA1 mCherry nor hM4D animals showed object bias in the sample phase (Fig. 2*B*). During the test phase, while iCA1 mCherry animals spent more time exploring the displaced object, hM4D animals explored both objects equally [Fig. 2*C*; two-way ANOVA, significant treatment × (nondisplaced vs displaced) object interaction: *F*_(1,15)_ = 8.869, *p* = 0.0094; no main effect of object: *F*_(1,15)_ = 0.534, *p* = 0.457 or of treatment: *F*_(1,15)_ = 0.251, *p* = 0.624; Sidak's post hoc test; mCherry: *t*_(15)_ = 2.728, *p* = 0.0309, hM4D: *t*_(15)_ = 1.521, *p* = 0.276]. Consistent with this, iCA1 hM4D animals showed a lower DI compared with mCherry (Fig. 2*D*; unpaired *t* test *t*_(15)_ = 2.985, *p* = 0.0093), and only the mCherry group exhibited a DI greater than chance level (one-sample *t* test; mCherry: *t*_(8)_ = 2.786, *p* = 0.0237; hM4D: *t*_(7)_ = 1.485, *p* = 0.181). Importantly, iCA1 mCherry and hM4D animals did not differ in their total exploration time (unpaired *t* test: *t*_(16)_ = 2.020, *p* = 0.0604) or distance traveled (unpaired *t* test: *t*_(16)_ = 0.878, *p* = 0.393) during the test phase, suggesting that this effect was not driven by changes in motor behavior or motivation. These data show that chemogenetic inactivation of iCA1 impairs OiP recognition memory.

10.1523/ENEURO.0105-26.2026.f2-1 Figure 2-1 **Viral expression maps of iCA1 manipulations**. Diagrams depicting overlaid viral spread for all iCA1 hM4D mice that underwent the OiP task in the allocentric (**A**) or egocentric (**B**) configurations. Download Figure 2-1, TIF file.

10.1523/ENEURO.0105-26.2026.f2-2 Figure 2-2 **iCA1 activity is not necessary for egocentric two-object OiP memory.** iCA1 manipulation during egocentric two-object OiP. **A**. Schematic of the task design for the egocentric version of the two-object OiP task. Animals underwent identical procedures as in Figure 1A, except that during the test phase they were placed in the chamber facing the same wall as the sample phase (depicted with an S in the scheme). **B**. mCherry and hM4D iCA1 mice showed no object preference during the sample phase (two-way ANOVA, no treatment x object type interaction: *F*_(1,24)­_ = 1.116, *p* = 0.3013; main effect of object: *F*_(1,24)_ = 2.311, *p* = 0.1514 or of treatment: *F*_(1,24)_ = 0.02565, *p* = 0.8741). **C**. During the test phase, hM4D iCA1 egocentric mice spent more time exploring the displaced (D) compared to the non-displaced (ND) objects. **D**. Calculation of a discrimination index for each animal showed that both mCherry and hM4D iCA1 egocentric animals displayed a preference for the displaced object vs chance levels. mCherry: n = 13 (6 females, 7 males); hM4D: n = 13 (6 females, 7 males). Individual datapoints from female mice are depicted as circles and male mice as squares for transparency, but no sex differences were found. *p < 0.05. Download Figure 2-2, TIF file.

To replicate previous findings in rats showing that the HPC is not required for the two-object OiP task when run in an egocentric configuration (Langston and Wood, 2010), we ran a control experiment in which we inhibited the iCA1 during egocentric two-object OiP task (Extended Data Fig. 2-2*A*). In the egocentric version of the task, mice are placed in the same location (facing the south wall) at the start of the sample and test phases (Extended Data Fig. 2-2*A*). iCA1 inhibition did not affect preference for the displaced object In the egocentric version of the OiP task, with iCA1 hM4D animals showing discrimination [Extended Data Fig. 2-2*B*: two-way ANOVA, main effect of object: *F*_(1,24)_ = 20.63, *p* = 0.0001; but no effect of treatment: *F*_(1,24)_ = 3.411, *p* = 0.560 or treatment × (nondisplaced vs displaced) object interaction: *F*_(1,24)_ = 3.411, *p* = 0.0771; Extended Data Fig. 2-2*C*: unpaired *t* test mCherry vs hM4D: *t*_(24)_ = 1.822, *p* = 0.0809; chance level comparison: one-sample *t* test; mCherry: *t*_(12)_ = 2.921, *p* = 0.0128, hM4D: *t*_(12)_ = 3.601, *p* = 0.0036]. We validated our C21-mediated inhibition of the iCA1 using slice electrophysiology (Fig. 2*E*,*F*: paired *t* test: *t*_(5)_ = 3.854, *p* = 0.012; Fig. 2*G*: paired *t* test: *t*_(5)_ = 3.245, *p* = 0.0228). Together, these data show that iCA1 activity is necessary for successful OiP recognition memory in the two-object allocentric, but not egocentric version of the OiP task in mice.

### Chemogenetic inhibition of the mPFC or the iCA1-mPFC pathway does not affect two-object OiP memory

**Figure 3.**
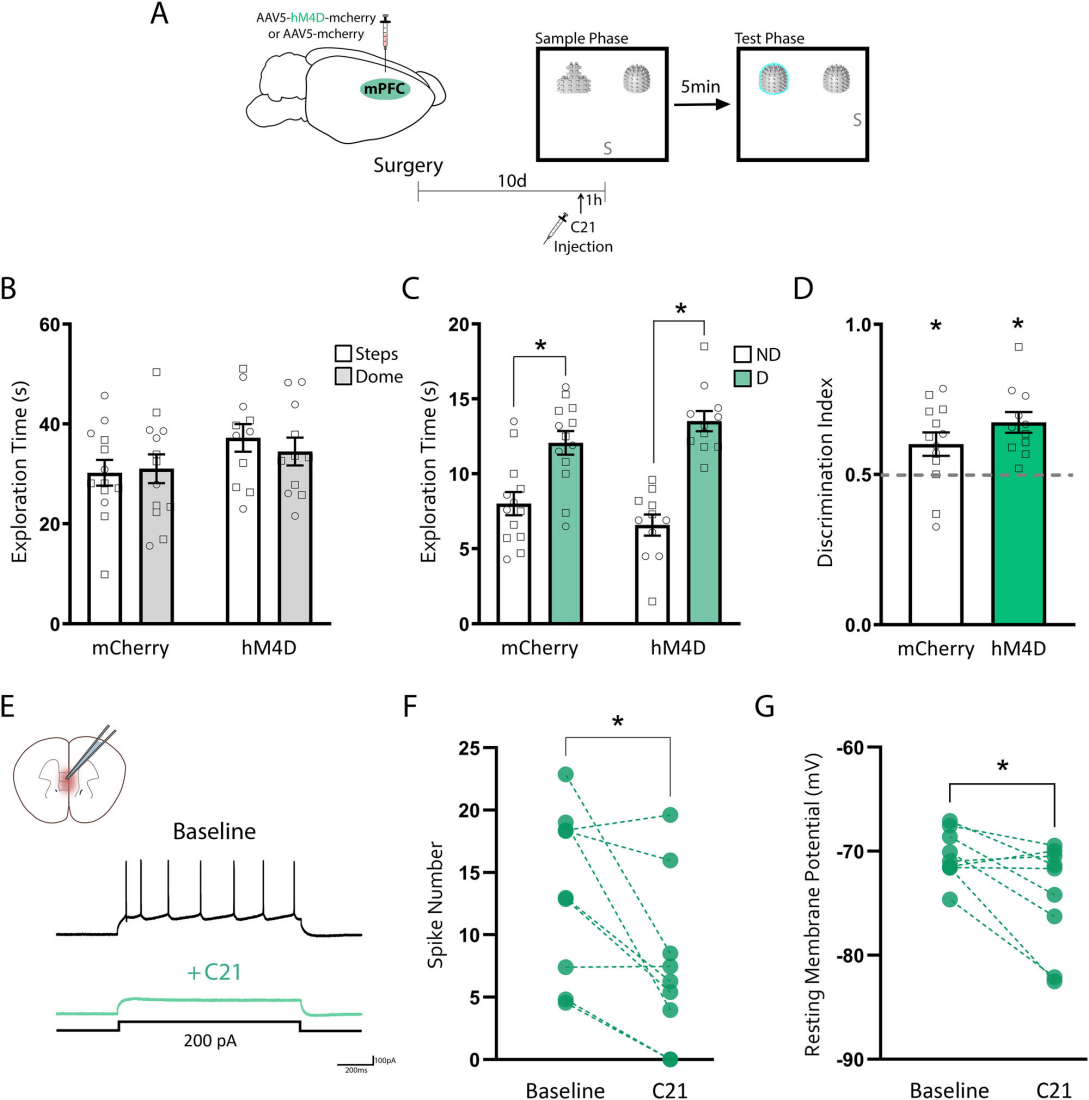
mPFC activity is not necessary for successful two-object OiP memory. ***A***, Experimental timeline. C57BL/6J mice were infused with AAV5-hM4D-mCherry or control AAV5-mCherry into the medial prefrontal cortex (mPFC). Ten days later, mice were injected with the DREADD agonist C21 1 h prior to testing in the allocentric version of the two-object OiP task. ***B***, mCherry and hM4D mPFC mice showed no object preference during the sample phase (two-way ANOVA, no treatment × object type interaction: *F*_(1,22)_ = 0.888, *p* = 0.3563; main effect of object: *F*_(1,22)_ = 0.2566, *p* = 0.6175 or of treatment: *F*_(1,22)_ = 2.312, *p* = 0.1426). ***C***, During the test phase, both mCherry and hM4D mPFC mice spent more time exploring the displaced (***D***) compared with the nondisplaced (ND) objects. ***D***, Calculation of a DI for each animal showed that both mCherry and hM4D mPFC animals displayed a preference for the displaced object. mCherry: *n* = 13 (5 females, 8 males); hM4D: *n* = 11 (5 females, 6 males). Individual datapoints from female mice are depicted as circles and male mice as squares for transparency, but no sex differences were found. ***E–G***, Electrophysiological validation of mPFC DREADD experiments. ***E***, Representative trace of action potentials recorded from a hM4D-mCherry+ neuron in the mPFC layer 5 at baseline and after application of C21. ***F***, The number of action potentials evoked by somatic current injection (100 pA) in hM4D+ transfected neurons was significantly decreased following bath application of C21. ***G***, The resting membrane potential (RMP) of hM4D + neurons became significantly more hyperpolarized following bath application of C21. (*n* = 9 cells, 6 mice). *p < 0.05. Extended Data Figure 3-1 shows mPFC viral target maps.

Substantial research implicates the mPFC in four-object OiP memory in rats (Barker and Warburton, 2008, 2009, 2011a, 2015; Savalli et al., 2015; Benn et al., 2016; Barker et al., 2017, 2020; Sabec et al., 2018), suggesting that this region might be similarly recruited for two-object OiP in mice. To test that, we infused the mPFC of mice with either control AAV5-mCherry or AAV5-hM4D-mCherry into the mPFC (see Extended Data Fig. 3-1 for viral targeting maps) and tested them 10 d later on the allocentric version of the two-object OiP task following C21 treatment (Fig. 3*A*). Animals showed no bias for object type during the sample phase (Fig. 3*B*). During the test phase, both mPFC mCherry and hM4D, animals showed a preference for the displaced object (Fig. 3*C*; two-way ANOVA, significant main effect of object: *F*_(1,22) _= 26.93, *p* < 0.0001, no treatment × object type interaction: *F*_(1,22)_ = 1.840, *p* = 0.189, or effect of treatment: *F*_(1,22) _= 0.0228, *p* = 0.881; Fig. 3*D*; unpaired *t* test: *t*_(22)_ = 1.363, *p* = 0.187; comparison to chance: one-sample *t* test; mCherry: *t*_(12)_ = 2.589, *p* = 0.0237; hM4D: *t*_(10)_ = 5.022, *p* = 0.0005), indicating that mPFC inactivation does not affect OiP recognition memory in the two-object, allocentric version of the OiP. Validation experiments using patch-clamp recordings confirmed that C21 triggered a reduction in spike number (Fig. 3*E–G*; paired *t* test: *t*_(8)_ = 3.157, *p* = 0.0135) and hyperpolarization (Fig. 3*G*; paired *t* test: *t*_(8)_ = 2.771, *p* = 0.0242) of mPFC pyramidal neurons expressing hM4D.

10.1523/ENEURO.0105-26.2026.f3-1 Figure 3-1 **Viral expression maps of mPFC manipulations**. Diagrams depicting overlaid viral spread for all mPFC hM4D infused animals. PL = Prelimbic cortex, IL = Infralimbic cortex, DP = Dorsal peduncular Cortex, DTT = Dorsal tenia tecta. Download Figure 3-1, TIF file.

**Figure 4.**
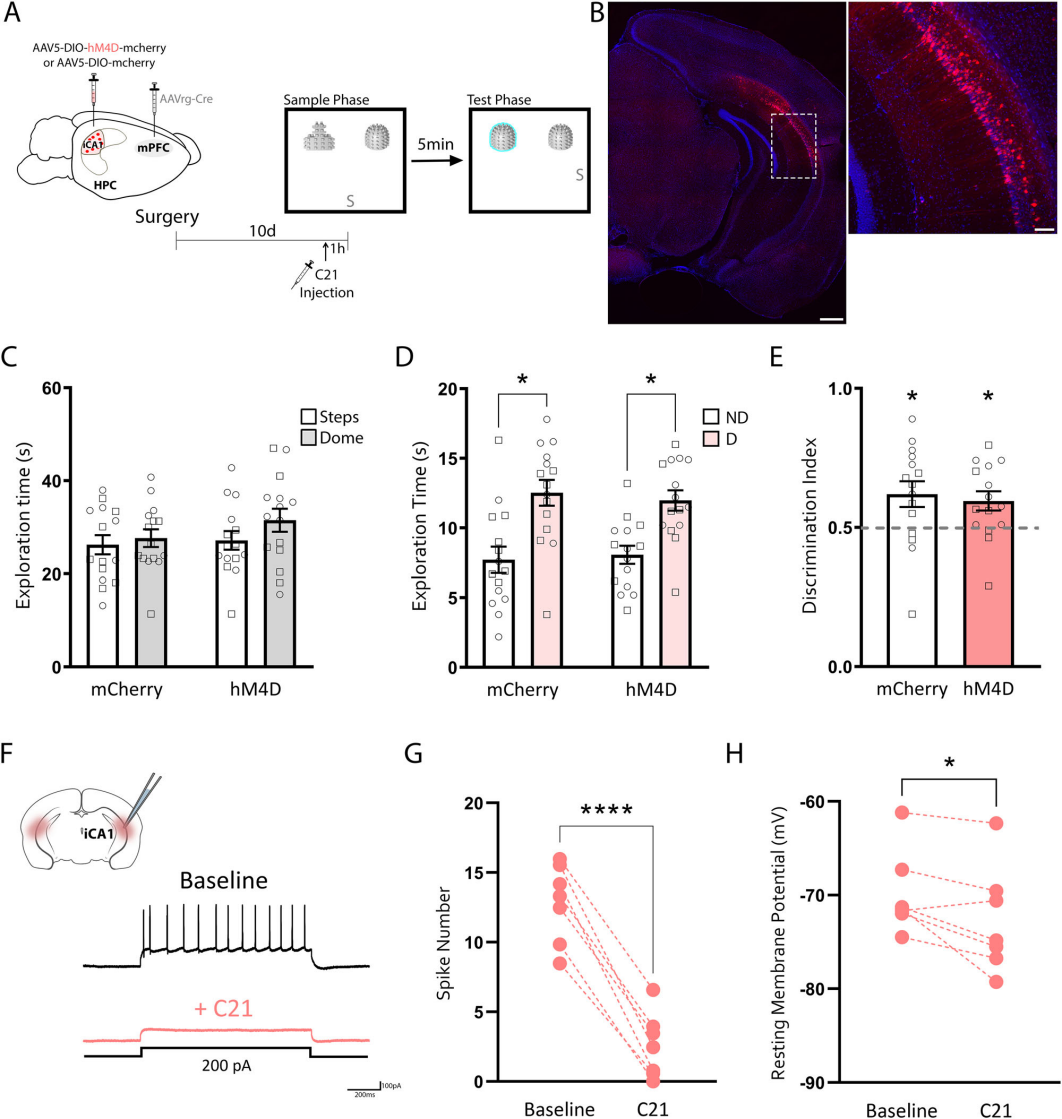
iCA1-mPFC activity is not necessary for successful two-object OiP memory. ***A***, Experimental timeline. C57BL/6J mice were infused with AAVrg-Cre into the medial prefrontal cortex (mPFC) and with either AAV5-DIO-hM4D-mCherry or control AAV5-DIO-mCherry into the intermediate CA1 (iCA1). Ten days later, mice were injected with the DREADD agonist C21 1 h prior to testing in the allocentric version of the two-object OiP task. ***B***, Representative image showing transfected cells (red) in the iCA1, with insert magnified on the right image. Scale bars are 500 µm (left) and 100 µm (right). ***C***, mCherry and hM4D iCA1-mPFC mice showed no object preference during the sample phase (two-way ANOVA, no treatment × object type interaction: *F*_(1,28)_ = 0.5907, *p* = 0.4486; main effect of object: *F*_(1,28)_ = 2.304, *p* = 0.1403 or of treatment: *F*_(1,28)_ = 1.060, *p* = 0.3121). ***D***, During the test phase, both mCherry and hM4D iCA1-mPFC mice spent more time exploring the displaced (***D***) compared with the nondisplaced (ND) objects. ***E***, Calculation of a DI for each animal showed that both mCherry and hM4D iCA1-mPFC animals displayed a preference for the displaced object. mCherry: *n* = 13 (5 females, 8 males); hM4D: *n* = 11 (5 females, 6 males). Individual datapoints from female mice are depicted as circles and male mice as squares for transparency, but no sex differences were found. ***F–H***, Electrophysiological validation of iCA1-mPFC DREADD experiments. ***F***, Representative trace of action potentials recorded from a mPFC-projecting iCA1 hM4D-mCherry+ neuron at baseline and after application of C21. ***G***, The number of action potentials evoked by somatic current injection (200 pA) in hM4D-mCherry+ transfected neurons was significantly decreased following bath application of C21 (*n* = 7 cells, 3 mice). ***H***, The resting membrane potential (RMP) of DIO-hM4D+ neurons became significantly more hyperpolarized following bath application of C21 (*n* = 7 cells, 3 mice). *p < 0.05, ****p < 0.0001.

These data suggest that the mPFC is not required for two-object OiP in mice. This is surprising given the impact of mPFC soma (Barker and Warburton, 2008, 2009, 2011a, 2015; Savalli et al., 2015; Benn et al., 2016; Barker et al., 2017, 2020; Sabec et al., 2018) and iCA1-mPFC (Barker et al., 2017) manipulations on four-object OiP tasks in rats. Nevertheless, given that select brain pathways may exert specialized functions not replicated by whole-region manipulations, we wanted to directly test whether iCA1-mPFC projections would be necessary for two-object OiP recognition memory in mice. We injected a retrograde virus expressing Cre recombinase (AAVrg-Cre) in the mPFC (spanning the infralimbic and prelimbic cortices) and virus expressing Cre-dependent hM4D (AAV5-DIO-hM4D-mCherry) or mCherry (AAV5-DIO-mCherry) in the iCA1 (Fig. 4*A*,*B*) to inhibit iCA1 neurons projecting to the mPFC. Mice were injected with C21 1 h prior to OiP training (Fig. 4*A*). We found no object bias (Fig. 4*C*) during the sample phase. During the test phase, both hM4D and mCherry mice spent more time exploring the displaced object (Fig. 4*D*; two-way ANOVA, significant main effect of object: *F*_(1,28)_ = 14.0, *p* = 0.0008, no effect of treatment: *F*_(1,28)_ = 1.591, *p* = 0.218 or treatment × object type interaction: *F*_(1,28)_ = 0.155, *p* = 0.697). Both groups demonstrated equal preference for the displaced object as measured by the DI (Fig. 4*E*; unpaired *t* test: *t*_(28)_ = 0.421, *p* = 0.677; comparison to chance: one-sample *t* test; mCherry: *t*_(14)_ = 2.579, *p* = 0.0219, hM4D: *t*_(14)_ = 2.743, *p* = 0.0159). Using whole-cell patch clamping to record from mCherry+ iCA1 pyramidal neurons of iCA1-mPFC hM4D mice (Fig. 4*F–H*), we confirmed that C21 bath application led to a reduction in spike number (Fig. 4*G*; paired *t* test: *t*_(6)_ = 12.90, *p* < 0.0001) and to hyperpolarization (Fig. 4*H*; paired *t* test: *t*_(6)_ = 2.704, *p* = 0.0354) of mPFC-projecting iCA1 pyramidal neurons expressing hM4D. These data suggest that iCA1-mPFC and mPFC activity are not necessary for the expression of two-object allocentric OiP memory in mice.

## Discussion

Here we tested the contribution of HPC subregions iCA1 and vCA1, the mPFC, and iCA1-mPFC connections to OiP memory in mice. We found that vCA1 and iCA1 activity is necessary for OiP recognition memory in the two-object allocentric OiP task with a short retention interval. In contrast, inhibition of mPFC soma or iCA1 neurons projecting to the mPFC did not affect OiP performance. Overall, our study is the first to establish a contribution of the vCA1 and iCA1 HPC subregions to OiP memory in mice, broadening our understanding of hippocampal modulation of spatial and recognition memory.

We found that either vCA1 or iCA1 inhibition was sufficient to impair two-object OiP memory. While several studies implicate the whole HPC in four-object (Liu and Bilkey, 2001; Good et al., 2007; Langston et al., 2010a; Barker and Warburton, 2011a;  Barker et al., 2017) and two-object (Langston and Wood, 2010) OiP memory, an effect replicated in monkeys (Bachevalier and Nemanic, 2008), far less is known about HPC subregion modulation of this type of recognition memory. Barker and Warburton showed that dCA1 inhibition impairs four-object OiP in rats (Barker and Warburton, 2015), similar to earlier findings implicating dCA1 (as well as dCA3 and dDG) in spatial recognition memory (OiP and object–location combined; Lee et al., 2005; Hunsaker et al., 2007). Barker and colleagues further reported a contribution of iCA1 (via its projections to mPFC) to four-object OiP recognition memory in rats (Barker et al., 2017). Even though ventral HPC is generally believed to be less involved in spatial processing compared with dorsal HPC (Nadel, 1968; Hock and Bunsey, 1998; Moser and Moser, 1998; Bannerman et al., 1999, 2004; Strange et al., 2014; but see de Hoz et al., 2003), our findings align with others showing vCA1 contribution to spatial recognition memory, particularly in the object–location task (Beer et al., 2014; Lin et al., 2021), with further indication of vCA1 contributing to temporal order (Hunsaker et al., 2008) and novel object (Neugebauer et al., 2018; but see Taylor et al., 2025) recognition memory. Together with our findings, this literature suggests a broader role for vCA1 in spatial processing, encompassing object–location and location-identity object recognition. Interestingly, while we recently reported a differential modulation of cognitive flexibility and OiP memory following developmental inhibition of iCA1 and vCA1 projections (Cruz-Sanchez et al., 2026), our data points to equivalent effects of either iCA1 or vCA1 acute soma inhibition on OiP memory in adult mice.

Our iCA1 soma manipulation data generally fits with the proposal by Barker and colleagues that intermediate HPC is recruited in recognition tasks that involve spatial “what–where” components (Barker et al., 2017), adding to an emerging literature on intermediate HPC (iHPC) function. The iHPC differs from its dorsal and ventral subdivisions in terms of cell population (Thompson et al., 2008; Cembrowski et al., 2016; Jin and Lee, 2021; Jarzebowski et al., 2022), efferent/afferent patterns (Fanselow and Dong, 2010), and synaptic activity (Izaki et al., 2001, 2002; Kawashima et al., 2006; Takita et al., 2010). Very few studies examined the functional role of the iHPC in memory and behavior. iHPC lesions are linked to deficits in one-trial spatial learning (Bast et al., 2009), with iCA1 activity contributing to the encoding of reward-location associations and to correlating changes in motivational significance with spatial information (Jin and Lee, 2021; Jarzebowski et al., 2022). As a whole, these data point to an important contribution of the iHPC in the encoding of spatial location associations, particularly in the integration of spatial location with emotional and motivational information, assisting goal-directed navigation (Jin and Lee, 2021; Jarzebowski et al., 2022). Importantly, we saw a dissociation for the requirement of iCA1 between allocentric and egocentric configurations of the two-object OiP task, consistent with work in rats showing that egocentric two-object OiP task does not rely on the HPC (Langston and Wood, 2010). While the spatial complexity of four-object OiP clearly supports a need for the HPC in its completion (Langston et al., 2010a; Barker and Warburton, 2011a; Barker et al., 2017), these data in rats and mice reinforce the need for careful consideration of experimental parameters when running the two-object version of the OiP task.

In contrast to the findings by Barker et al. (2017), we found that the iCA1-mPFC pathway is not necessary for two-object OiP performance in mice. The iCA1 sends strong unidirectional projections to the prelimbic and infralimbic subdivisions of the mPFC (Takita et al., 2013), and the iHPC-mPFC pathway has been implicated in working memory (Izaki et al., 2008) and valence changes in spatial navigation (Blanquat et al., 2013). Furthermore, OiP learning increases synchronization and theta phase locking between CA1 and mPFC in rats (Kim et al., 2011), with HPC-mPFC synaptic plasticity mechanisms being linked to OiP performance (Sabec et al., 2018), emphasizing a role for HPC-mPFC projections in OiP memory processing in rats. Perhaps even more surprisingly, mPFC inhibition also failed to affect two-object OiP performance in mice. Lesion, pharmacological, and optogenetic mPFC loss- (Barker and Warburton, 2008; Cross et al., 2013; Savalli et al., 2015) and gain-of-function (Benn et al., 2016) manipulations in rats impair and facilitate four-object OiP memory at 5 min retention intervals, respectively, pointing to a robust contribution of the mPFC to four-object OiP at short delays. Technical, species, or experimental design (four- vs two-object) differences might be responsible for our negative results of iCA1-mPFC and mPFC inhibition. It is important to note that while both the four- and two-object versions of the OiP tests are often treated as indistinctive assessments of OiP memory in the rodent literature, the question of whether they rely on the same brain network has not been directly tested. Additionally, studies suggest that mPFC activity is greater when spatial memory tasks are run in an egocentric configuration (De Bruin et al., 2001; Nieto-Escámez et al., 2002; Ma et al., 2003), raising the question of whether egocentric two-object OiP might require the mPFC.

Could another iCA1 downstream target mediate two-object OiP memory in mice? The perirhinal cortex (PRh; Bussey et al., 2000; Barker and Warburton, 2008, 2009; Brown et al., 2012; Barker et al., 2017) and its connections with the HPC (Barker and Warburton, 2011a) are necessary for four-object OiP expression, as well as for OiP memory tasks using the Y maze in rats (Bussey et al., 2001) and OiP visual tasks in monkeys (Bachevalier and Nemanic, 2008). Interestingly, inhibition of the PRh during a two-object, radial maze OiP paired-associate task led to reduced dorsal CA1 activity (Lee and Park, 2013), suggesting that iCA1-PRh activity might be similarly affected in two-object OiP. However, the PRh only receives sparse projections from the iCA1 (Cenquizca and Swanson, 2007; Agster and Burwell, 2013), and a functional role for the iCA1-PRh pathway has not been described. In contrast, the iCA1 sends strong projections to the nucleus accumbens (NAc; Kelley and Domesick, 1982; Groenewegen et al., 1999) and nucleus reuniens (Re; McKenna and Vertes, 2004), two brains regions also implicated in OiP memory expression (Roullet et al., 2001; Barker and Warburton, 2018). Overall, while recent studies have made significant progress in elucidating specialized functions for the iCA1, the broader functional contribution of its downstream partners to behavior remains unknown. Similarly, future work will need to elucidate whether our effects of vCA1 inhibition are mediated by its projections to mPFC or other downstream targets. Overall, this study identified a novel role for the vCA1 and iCA1 in two-object OiP recognition memory in mice, adding to a growing literature showing specialized hippocampal subregion contributions to spatial memory. Our findings specify the function of select HPC subregions in “what–where” associations in mice, deepening our understanding of the neural signatures of spatial recognition processing.

## Synthesis

Reviewing Editor: Mihaela Iordanova, Concordia University - Loyola Campus Decisions are customarily a result of the Reviewing Editor and the peer reviewers coming together and discussing their recommendations until a consensus is reached. When revisions are invited, a fact-based synthesis statement explaining their decision and outlining what is needed to prepare a revision will be listed below. The following reviewer(s) agreed to reveal their identity: Patrese robinsondrummer. Note: If this manuscript was transferred from JNeurosci and a decision was made to accept the manuscript without peer review, a synthesis may not be available.

The manuscript by Cruz-Sanchez and colleagues employed a chemogenetics approach to investigate the role of the ventral and intermediate CA1 subregions (vCA1 and iCA1, respectively) of the hippocampus, medial prefrontal cortex (mPFC) and iCA1-mPFC pathway in a two-object version of the object-in-place (OiP) recognition memory task in mice. The authors found that vCA1 and iCA1, but not mPFC and iCA1-mPFC pathway, are necessary for allocentric two-object OiP memory. Overall, this is a novel and interesting study.

The reviewers noted the manuscript is well-written, and the experiments are thoughtfully designed, executed, and appropriately analyzed (but see below) with adequate sample sizes across groups. Both reviewers have only a few minor, mostly methodological clarifications or suggestions, which do not detract from the overall impact and significance of the findings.

The authors describe using an unpaired t-test to examine discrimination index (DI) scores in each group. However, because the DI score is compared with a hypothetical chance score (0.5) rather than with an independent second group of animals, the unpaired t-test is inappropriate. Rather, a one-sample t-test comparing each group to chance is the appropriate statistical test. Unless the authors mean to say that the group DI was compared to another group of chance DI values?

Figures 1 and 2 have legends in the caption for panels I-k however the lettering in the figure panel only goes to letter G. Relabeling is needed.

Line 125: Please spell out what iCA1 means in the Introduction.

Line 174: Please clarify whether the objects used were also cleaned with water or 70% ethanol between phases and subject.

Line 184: It would be informative to have a more detailed explanation of the test phase. That is, the reasoning behind placing two copies of the same object in the test phase is unclear in this section.

Line 186- It would be helpful here to say which experiments have egocentric and which have allocentric results, and why you chose to have one or the other for the given brain region.

Lines 204-206 As it is written, it was difficult to understand exactly how behavior was measured. The animals underwent a 3min test phase, but was only the first 20s of the phase scored? Or was the discrimination ratio determined only after the animals accumulated 20s of object exploration between the two objects?

Line 267: Please spell out what DI means in the Statistical Analysis section or define it in line 201.

Lines 309-313: This detailed explanation would be more beneficial and better placed in the methods section to address the above comment 3.

Line 336-It is difficult to understand what was measured here. Is this the total exploration time (time with ND +time with D) compared between each group? If so, that should be stated somewhere and perhaps add "total" before "exploration time" so that it is clear that this is different than what is being displayed in Figure 1C.

Line 239-Can you mention how long after testing the animals were euthanized?

Line 852: Please clarify how the discrimination index was calculated.

## Author Response

We thank the reviewers and the reviewing editor for their time and thoughtful analysis of our manuscript. We have incorporated their excellent suggestions, which corrected and clarified several aspects of our submission and overall made the manuscript much stronger. Below is a point-by-point reply to all comments (line numbers reference the manuscript file with tracked changes):

The authors describe using an unpaired t-test to examine discrimination index (DI) scores in each group. However, because the DI score is compared with a hypothetical chance score (0.5) rather than with an independent second group of animals, the unpaired t-test is inappropriate. Rather, a one-sample t-test comparing each group to chance is the appropriate statistical test. Unless the authors mean to say that the group DI was compared to another group of chance DI values? We thank the reviewers for identifying this error. We have corrected the statistical analyses of discrimination index comparison to chance to one-sample t test (lines 283, 349-350, 379-380, 395-396, 415-416, 437-438). There were no changes in significance.

Figures 1 and 2 have legends in the caption for panels I-k however the lettering in the figure panel only goes to letter G. Relabeling is needed.

We thank the reviewers for catching this mistake. We have corrected this in Figure 1 and Figure 2 legends (lines 877-881; 900-904).

Line 125: Please spell out what iCA1 means in the Introduction.

We have corrected this. Line 125 now reads: "Given reports implicating intermediate CA1 (iCA1)-mPFC projections (Barker et al., 2017b) (...)" Line 174: Please clarify whether the objects used were also cleaned with water or 70% ethanol between phases and subject.

We have clarified that objects were also cleaned with water between subjects and with 70% ethanol at the end of the day. Line 174 now reads: "Behavioral chambers and objects were cleaned with water between phases and subjects, and with 70% ethanol at the end of the day." Line 184: It would be informative to have a more detailed explanation of the test phase. That is, the reasoning behind placing two copies of the same object in the test phase is unclear in this section.

We thank the reviewers for highlighting the need for clarification. We have expanded this section. It now reads (from line 179): "In the 5-minute sample phase (Davis et al., 2013), mice interacted with two objects (dome and step), after which animals were removed and placed back into their home cage. Object identity and location combinations were counterbalanced across animals. After a 5-minute delay (Davis et al., 2013; Bonardi et al., 2021), animals underwent a 3-minute test phase (Davis et al., 2013) in which they were placed in the chamber with two copies of the same object (two domes or two steps). Given that in the test phase the two objects are familiar and identical, the test measures novelty via identification of the novel object-location contingency (i.e. the copy of the dome object that is positioned where the step object had been, or vice-versa).

Line 186- It would be helpful here to say which experiments have egocentric and which have allocentric results, and why you chose to have one or the other for the given brain region.

We have added this information. From line 188, the revised manuscript now reads: "This task was run in two versions: an egocentric or an allocentric version (Langston and Wood, 2010; Davis et al., 2013), which varied in the placement of the mouse at the start of the test phase to foster different spatial exploration strategies. All experiments were run in the allocentric version except for the iCA1 inhibition in Extended Data Figure 2-2, a control experiment used to confirm that the hippocampus is not required for egocentric two-object OiP (Langston and Wood, 2010)." Lines 204-206 As it is written, it was difficult to understand exactly how behavior was measured. The animals underwent a 3min test phase, but was only the first 20s of the phase scored? Or was the discrimination ratio determined only after the animals accumulated 20s of object exploration between the two objects? We agree this was confusing in the manuscript, and have clarified it in the text. The animals underwent a 3-minute test phase, but only the first 20 seconds of total object exploration were analyzed, a recommendation made by Leger et al., 2013 (adapted from Arqué et al., 2008), which we have used in our previous object recognition papers. Our results using the 20-second criterion do not differ from those analyzing the full 3-minutes of the task, but maintain consistency across our publications and datasets, thus easing comparisons across tasks.

This section now reads (from line 202): "Behavior was analyzed using ANY-maze® software (Stoelting). Exploratory activity was defined as in Leger et al., (Leger et al., 2013) (Cruz-Sanchez et al., 2020). Briefly, object exploration was defined as an object-directed gaze while actively sniffing and/or pawing within 2 cm of the object. Sitting on top of the object while sniffing the surrounding air or chewing the object were not considered exploration. Following Leger et al.'s (Leger et al., 2013) recommendation [adapted from (Arqué et al., 2008)], the analysis of object exploration in the test phase of all experiments comprised only the first 20 seconds of total interaction time with the objects (Cruz-Sanchez et al., 2020). This is further supported by studies showing rodents demonstrate a higher preference for the novelty within the first 60 to 120 seconds of the test phase (Dix and Aggleton, 1999; Mumby, 2001; Mumby et al., 2002; Cruz-Sanchez et al., 2020), which corresponds to when mice reached the 20-second criterion in our sample. A discrimination index (DI) was calculated as a measure of relative novelty preference by dividing the amount of time spent exploring the displaced object by the total time spent exploring both the displaced and non-displaced objects.

Line 267: Please spell out what DI means in the Statistical Analysis section or define it in line 201.

We have done both. We have introduced the DI acronym in line 213 (see above) and spelled it out in lines 281-282: "A discrimination index (DI) comparison between treatment groups was made using an unpaired t-test." Lines 309-313: This detailed explanation would be more beneficial and better placed in the methods section to address the above comment 3.

We have clarified these experimental design details and choices in the methods section as suggested (from line 202, described above). Given that many readers skip the methods section, we have kept the original results section text to better orient the reader to the task design, but would be happy to remove it if the reviewers and reviewing editor believe it is unnecessary and/or repetitive.

Line 336-It is difficult to understand what was measured here. Is this the total exploration time (time with ND +time with D) compared between each group? If so, that should be stated somewhere and perhaps add "total" before "exploration time" so that it is clear that this is different than what is being displayed in Figure 1C.

We thank the reviewers for bringing this to our attention. We have clarified this from line 350: "vCA1 mCherry and hM4D animals displayed equivalent total exploration time (total time spent exploring the displaced and non-displaced objects across the 3-minute session; unpaired t-test: t(18)=0.1198, p=0.906)".

Line 239-Can you mention how long after testing the animals were euthanized? Mice underwent slice electrophysiology as early as 3 days after testing. Due to serious personnel and resource constraints, a portion of the animals in the iCA1-mPFC cohort was delayed in their patching, but we saw no difference in C21 efficiency between the short and long-interval animals. We have added information on this (also acknowledging the delay) in lines 250-252.

Line 852: Please clarify how the discrimination index was calculated.

We have clarified this in the revised manuscript. The legend for Figure 1D now reads (from line 871-873): "D. A discrimination index (calculated by dividing the amount of time spent exploring the displaced object by the total time spent exploring both the displaced and non-displaced objects) for each animal showed that hM4D vCA1 animals did not display a preference for the displaced object."

## References

[B1] Agster KL, Burwell RD (2013) Hippocampal and subicular efferents and afferents of the perirhinal, postrhinal, and entorhinal cortices of the rat. Behav Brain Res 254:50–64. 10.1016/j.bbr.2013.07.005 23872326 PMC3792719

[B3] Ainge JA, Langston RF (2012) Ontogeny of neural circuits underlying spatial memory in the rat. Front Neural Circuits 6:1–10. 10.3389/fncir.2012.00008 22403529 PMC3290765

[B2] Ainge JA, Langston RF, Ascoli G, Mason G (2012) Ontogeny of neural circuits underlying spatial memory in the rat. Front Neural Circuits 6:1–10. 10.3389/fncir.2012.00008 22403529 PMC3290765

[B4] Ameen-Ali KE, Easton A, Eacott MJ (2015) Moving beyond standard procedures to assess spontaneous recognition memory. Neurosci Biobehav Rev 53:37–51. 10.1016/j.neubiorev.2015.03.013 25842032

[B5] Arqué G, Fotaki V, Fernández D, de Lagrán MM, Arbonés ML, Dierssen M (2008) Impaired spatial learning strategies and novel object recognition in mice haploinsufficient for the dual specificity tyrosine-regulated kinase-1A (Dyrk1A). PLoS One 3:e2575. 10.1371/journal.pone.0002575 18648535 PMC2481280

[B7] Arruda-Carvalho M, Clem RL (2014) Pathway-selective adjustment of prefrontal-amygdala transmission during fear encoding. J Neurosci 34:15601–15609. 10.1523/JNEUROSCI.2664-14.2014 25411488 PMC6608438

[B6] Arruda-Carvalho M, Wu WC, Cummings KA, Clem RL (2017) Optogenetic examination of prefrontal-amygdala synaptic development. J Neurosci 37:2976–2985. 10.1523/JNEUROSCI.3097-16.2017 28193691 PMC5354336

[B8] Bachevalier J, Nemanic S (2008) Memory for spatial location and object-place associations are differently processed by the hippocampal formation, parahippocampal areas TH/TF and perirhinal cortex. Hippocampus 18:64–80. 10.1002/hipo.20369 17924520

[B9] Ballendine SA, Greba Q, Dawicki W, Zhang X, Gordon JR, Howland JG (2015) Behavioral alterations in rat offspring following maternal immune activation and ELR-CXC chemokine receptor antagonism during pregnancy: implications for neurodevelopmental psychiatric disorders. Prog Neuro-Psychopharmacol Biol Psychiatry 57:155–165. 10.1016/j.pnpbp.2014.11.002 PMC4464825 25445065

[B10] Bannerman DM, Yee B, Good M, Heupel M, Iversen S, Rawlins JNP (1999) Double dissociation of function within the hippocampus : a comparison of dorsal, ventral, and complete hippocampal cytotoxic lesions. Behav Neurosci 113:1170–1188. 10.1037/0735-7044.113.6.1170 10636297

[B11] Bannerman DM, Rawlins JNP, McHugh SB, Deacon RMJ, Yee BK, Bast T, Zhang WN, Pothuizen HHJ, Feldon J (2004) Regional dissociations within the hippocampus - memory and anxiety. Neurosci Biobehav Rev 28:273–283. 10.1016/j.neubiorev.2004.03.004 15225971

[B15] Barker GRI, Warburton EC (2008) NMDA receptor plasticity in the perirhinal and prefrontal cortices is crucial for the acquisition of long-term object-in-place associative memory. J Neurosci 28:2837–2844. 10.1523/JNEUROSCI.4447-07.2008 18337414 PMC6670687

[B16] Barker GRI, Warburton EC (2009) Critical role of the cholinergic system for object-in-place associative recognition memory. Learn Mem 16:8–11. 10.1101/lm.1121309 19117911 PMC2632853

[B17] Barker GRI, Warburton EC (2011a) When is the hippocampus involved in recognition memory? J Neurosci 31:10721–10731. 10.1523/JNEUROSCI.6413-10.2011 21775615 PMC6622630

[B18] Barker GRI, Warburton EC (2011b) Evaluating the neural basis of temporal order memoryfor visual stimuli in the rat. Eur J Neurosci 33:705–716. 10.1111/j.1460-9568.2010.07555.x 21226775

[B19] Barker GRI, Warburton EC (2015) Object-in-place associative recognition memory depends on glutamate receptor neurotransmission within two defined hippocampal-cortical circuits: a critical role for AMPA and NMDA receptors in the hippocampus, perirhinal, and prefrontal cortices. Cereb Cortex 25:472–481. 10.1093/cercor/bht245 24035904 PMC4380082

[B20] Barker GRI, Warburton EC (2018) A critical role for the nucleus reuniens in long-term, but not short-term associative recognition memory formation. J Neurosci 38:3208–3217. 10.1523/JNEUROSCI.1802-17.2017 29449430 PMC6596056

[B21] Barker GRI, Warburton EC (2020) Putting objects in context: a prefrontal–hippocampal–perirhinal cortex network. Brain Neurosci Adv 4:1–11. 10.1177/2398212820937621 PMC7479864 32954004

[B12] Barker GRI, Bird F, Alexander V, Warburton EC (2007) Recognition memory for objects, place, and temporal order: a disconnection analysis of the role of the medial prefrontal cortex and perirhinal cortex. J Neurosci 27:2948–2957. 10.1523/JNEUROSCI.5289-06.2007 17360918 PMC6672574

[B13] Barker GRI, Banks PJ, Scott H, Ralph GS, Mitrophanous KA, Wong LF, Bashir ZI, Uney JB, Warburton EC (2017) Separate elements of episodic memory subserved by distinct hippocampal-prefrontal connections. Nat Neurosci 20:242–250. 10.1038/nn.4472 28067902

[B14] Barker GRI, Wong LF, Uney JB, Warburton EC (2020) CREB transcription in the medial prefrontal cortex regulates the formation of long-term associative recognition memory. Learn Mem 27:45–51. 10.1101/lm.050021.119 31949036 PMC6970425

[B22] Bast T, Wilson LA, Witter MP, Morris RGM (2009) From rapid place learning to behavioral performance: a key role for the intermediate hippocampus. PLoS Biol 7:0730–0746. 10.1371/journal.pbio.1000089 PMC2671558 19385719

[B23] Beer Z, Chwiesko C, Sauvage MM (2014) Processing of spatial and non-spatial information reveals functional homogeneity along the dorso-ventral axis of CA3, but not CA1. Neurobiol Learn Mem 111:56–64. 10.1016/j.nlm.2014.03.001 24657342

[B24] Benn A, Barker GRI, Stuart SA, Roloff EVL, Teschemacher AG, Clea Warburton E, Robinson ESJ (2016) Optogenetic stimulation of prefrontal glutamatergic neurons enhances recognition memory. J Neurosci 36:4930–4939. 10.1523/JNEUROSCI.2933-15.2016 27147648 PMC4854963

[B25] Bevins RA, Besheer J (2006) Object recognition in rats and mice : a one-trial non-matching-to-sample learning task to study ‘recognition memory. Nat Protocol 1:1306–1311. 10.1038/nprot.2006.205 17406415

[B26] Biane JS, et al. (2023) Neural dynamics underlying associative learning in the dorsal and ventral hippocampus. Nat Neurosci 26:798–809. 10.1038/s41593-023-01296-6 37012382 PMC10448873

[B27] Blanquat PDS, Hok V, Save E, Poucet B, Chaillan FA (2013) Differential role of the dorsal hippocampus, ventro-intermediate hippocampus, and medial prefrontal cortex in updating the value of a spatial goal. Hippocampus 23:342–351. 10.1002/hipo.22094 23460312

[B28] Bonardi C, Pardon MC, Armstrong P (2016) Deficits in object-in-place but not relative recency performance in the APPswe/PS1dE9 mouse model of Alzheimer’s disease: implications for object recognition. Behav Brain Res 313:71–81. 10.1016/j.bbr.2016.07.008 27395445

[B29] Bonardi C, Pardon MC, Armstrong P (2021) Time or place? Dissociation between object-in-place and relative recency in young APPswe/PS1dE9 mice. Behav Neurosci 135:39–50. 10.1037/bne0000431 33856843

[B30] Botterill JJ, Khlaifia A, Walters BJ, Brimble MA, Scharfman HE, Arruda-Carvalho M (2021) Off-target expression of Cre-dependent adeno- associated viruses in wild-type C57BL/6J mice. eNeuro 8:1–16. 10.1523/ENEURO.0363-21.2021 PMC8614227 34785571

[B32] Brown MW, Aggleton JP (2001) Recognition memory: what are the roles of the perirhinal cortex and hippocampus? Nat Rev Neurosci 2:51–61. 10.1038/35049064 11253359

[B31] Brown MW, Barker GRI, Aggleton JP, Warburton EC (2012) What pharmacological interventions indicate concerning the role of the perirhinal cortex in recognition memory. Neuropsychologia 50:3122–3140. 10.1016/j.neuropsychologia.2012.07.034 22841990 PMC3500694

[B33] Burglen F, Marczewski P, Mitchell KJ, van der Linden M (2004) Impaired performance in a working memory binding task in patients with schizophrenia. Psychiatry Res 125:247–255. 10.1016/j.psychres.2003.12.014 15051185

[B34] Bussey TJ, Duck J, Muir JL, Aggleton JP (2000) Distinct patterns of behavioural impairments resulting from fornix transection or neurotoxic lesions of the perirhinal and postrhinal cortices in the rat. Behav Brain Res 111:187–202. 10.1016/S0166-4328(00)00155-8 10840144

[B35] Bussey TJ, Dias R, Amin E, Muir JL, Aggleton JP (2001) Perirhinal cortex and place-object conditional learning in the rat. Behav Neurosci 115:776–785. 10.1037/0735-7044.115.4.776 11508717

[B36] Calamandrei G, Rufini O, Valanzano A, Puopolo M (2002a) Long-term effects of developmental exposure to zidovudine on exploratory behavior and novelty discrimination in CD-1 mice. Neurotoxicol Teratol 24:529–540. 10.1016/S0892-0362(02)00234-9 12127899

[B37] Calamandrei G, Valanzano A, Ricceri L (2002b) NGF induces appearance of adult-like response to spatial novelty in 18-day male mice. 136.

[B38] Castilla-Ortega E, Pedraza C, Chun J, de Fonseca FR, Estivill-Torrús G, Santín LJ (2012) Hippocampal c-Fos activation in normal and LPA 1-null mice after two object recognition tasks with different memory demands. Behav Brain Res 232:400–405. 10.1016/j.bbr.2012.04.018 22537775

[B39] Cembrowski MS, Bachman JL, Wang L, Sugino K, Shields BC, Spruston N (2016) Spatial gene-expression gradients underlie prominent heterogeneity of CA1 pyramidal neurons. Neuron 89:351–368. 10.1016/j.neuron.2015.12.013 26777276

[B40] Cenquizca LA, Swanson LW (2007) Spatial organization of direct hippocampal field CA1 axonal projections to the rest of the cerebral cortex. Brain Res Rev 56:1–26. 10.1016/j.brainresrev.2007.05.002 17559940 PMC2171036

[B41] Connors EJ, Shaik AN, Migliore MM, Kentner AC (2014) Brain, behavior, and immunity environmental enrichment mitigates the sex-specific effects of gestational inflammation on social engagement and the hypothalamic pituitary adrenal axis-feedback system. Brain Behav Immun 42:178–190. 10.1016/j.bbi.2014.06.020 25011058

[B42] Cross L, Brown MW, Aggleton JP, Warburton EC (2013) The medial dorsal thalamic nucleus and the medial prefrontal cortex of the rat function together to support associative recognition and recency but not item recognition. Learn Mem 20:41–50. 10.1101/lm.028266.112 PMC3533127 23263843

[B43] Cruz-Sanchez A, Dematagoda S, Ahmed R, Mohanathaas S, Odenwald N, Arruda-Carvalho M (2020) Developmental onset distinguishes three types of spontaneous recognition memory in mice. Sci Rep 10:25–26. 10.1038/s41598-020-67619-w 32606443 PMC7326931

[B44] Cruz-Sanchez A, Ladouceur KE, Abdusalom A, Cruz-Sanchez A, Ladouceur KE, Abdusalom A, Chasiotis H, Gugustea R (2026) Maturation of hippocampus-medial prefrontal cortex projections defines a pathway-specific sensitive period for cognitive flexibility. Cell Rep 45:116812. 10.1016/j.celrep.2025.116812 41533508

[B45] Davis KE, Easton A, Eacott MJ, Gigg J (2013) Episodic-like memory for what-where-which occasion is selectively impaired in the 3xTgAD mouse model of Alzheimer’s disease. J Alzheimer’s Dis 33:681–698. 10.3233/JAD-2012-121543 23034524

[B46] De Bruin JPC, Moita MP, De Brabander HM, Joosten RNJMA (2001) Place and response learning of rats in a Morris water maze: differential effects of fimbria fornix and medial prefrontal cortex lesions. Neurobiol Learn Mem 75:164–178. 10.1006/nlme.2000.3962 11222058

[B47] de Hoz L, Knox J, Morris RGM (2003) Longitudinal axis of the hippocampus : both septal and temporal poles of the hippocampus support water maze spatial learning depending on the training protocol. Hippocampus 603:587–603. 10.1002/hipo.10079 12921349

[B48] Dix SL, Aggleton JP (1999) Extending the spontaneous preference test of recognition: evidence of object-location and object-context recognition. Behav Brain Res 99:191–200. 10.1016/S0166-4328(98)00079-5 10512585

[B49] Dong HW, Swanson LW, Chen L, Fanselow MS, Toga AW (2009) Genomic-anatomic evidence for distinct functional domains in hippocampal field CA1. Proc Natl Acad Sci U S A 106:11794–11799. 10.1073/pnas.0812608106 19561297 PMC2710698

[B50] Eacott MJ, Norman G (2004) Integrated memory for object, place, and context in rats: a possible model of episodic-like memory? J Neurosci 24:1948–1953. 10.1523/JNEUROSCI.2975-03.2004 14985436 PMC6730393

[B51] Eichenbaum H (2001) The hippocampus and declarative memory: cognitive mechanisms and neural codes. Behav Brain Res 127:199–207. 10.1016/S0166-4328(01)00365-5 11718892

[B52] Eichenbaum H (2004) Hippocampus : cognitive processes and neural representations that underlie declarative memory the hippocampus serves a critical role in declarative. Neuron 44:109–120. 10.1016/j.neuron.2004.08.028 15450164

[B53] Fanselow MS, Dong H-W (2010) Are the dorsal and ventral hippocampus functionally distinct structures? Neuron 65:7–19. 10.1016/j.neuron.2009.11.031 20152109 PMC2822727

[B54] Franklin KBJ, Paxinos G (2007) *The mouse brain in stereotaxic coordinates*, Ed 3. Boston: Elsevier.

[B55] Gaskin S, Tremblay A, Mumby DG (2003) Retrograde and anterograde object recognition in rats with hippocampal lesions. Hippocampus 13:962–969. 10.1002/hipo.10154 14750658

[B56] Good MA, Barnes P, Staal V, McGregor A, Honey RC (2007) Context- but not familiarity-dependent forms of object recognition are impaired following excitotoxic hippocampal lesions in rats. Behav Neurosci 121:218–223. 10.1037/0735-7044.121.1.218 17324066

[B57] Granon S, Save E, Buhot MC, Poucet B (1996) Effortful information processing in a spontaneous spatial situation by rats with medial prefrontal lesions. Behav Brain Res 78:147–154. 10.1016/0166-4328(95)00242-1 8864046

[B58] Groenewegen HJ, Mulder AB, Beijer AVJ, Wright CI, Da Silva FH L, Pennartz CMA (1999) Hippocampal and amygdaloid interactions in the nucleus accumbens. Psychobiology 27:149–164. 10.3758/BF03332111

[B59] Hall JH, Wiseman FK, Fisher EMC, Tybulewicz VLJ, Harwood JL, Good MA (2016) Neurobiology of learning and memory Tc1 mouse model of trisomy-21 dissociates properties of short- and long-term recognition memory. Neurobiol Learn Mem 130:118–128. 10.1016/j.nlm.2016.02.002 26868479 PMC4898594

[B60] Hock BJ, Bunsey MD (1998) Differential effects of dorsal and ventral hippocampal lesions. J Neurosci 18:7027–7032. 10.1523/JNEUROSCI.18-17-07027.1998 9712671 PMC6792982

[B61] Howland JG, Cazakoff BN, Zhang Y (2012) Altered object-in-place recognition memory, prepulse inhibition, and locomotor activity in the offspring of rats exposed to a viral mimetic during pregnancy. Neuroscience 201:184–198. 10.1016/j.neuroscience.2011.11.011 22119062 PMC4464820

[B62] Hunsaker MR, Mooy GG, Swift JS, Kesner RP (2007) Dissociations of the medial and lateral perforant path projections into dorsal DG, CA3, and CA1 for spatial and nonspatial (visual object) information processing. Behav Neurosci 121:742–750. 10.1037/0735-7044.121.4.742 17663599

[B63] Hunsaker MR, Fieldsted PM, Rosenberg JS, Kesner RP (2008) Dissociating the roles of dorsal and ventral CA1 for the temporal processing of spatial locations, visual objects, and odors. Behav Neurosci 122:643–650. 10.1037/0735-7044.122.3.643 18513134

[B64] Inayat M, Cruz-Sanchez A, Thorpe HHA, Frie JA, Richards BA, Khokhar JY, Arruda-Carvalho M (2021) Promoting and optimizing the use of 3d-printed objects in spontaneous recognition memory tasks in rodents: a method for improving rigor and reproducibility. eNeuro 8:ENEURO.0319-21.2021. 10.1523/ENEURO.0319-21.2021 PMC8489023 34503967

[B65] Inayat M, Cruz-Sanchez A, LaDouceur K, Parikh P, Arruda-Carvalho M (2026) Task optimization and ontogenetic profile of object-in-place recognition memory in mice. *Biorxiv*. 10.1523/ENEURO.0105-26.2026 PMC13271815 42236229

[B66] Izaki Y, Takita M, Nomura M (2001) Mouse hippocampo-prefrontal paired-pulse facilitation and long-term potentiation in vivo. Neuroreport 12:1191–1193. 10.1097/00001756-200105080-00028 11338190

[B67] Izaki Y, Takita M, Nomura M (2002) Local properties of CA1 region in hippocampo-prefrontal synaptic plasticity in rats. Neuroreport 13:469–472. 10.1097/00001756-200203250-00022 11930163

[B68] Izaki Y, Takita M, Akema T (2008) Specific role of the posterior dorsal hippocampus-prefrontal cortex in short-term working memory. Eur J Neurosci 27:3029–3034. 10.1111/j.1460-9568.2008.06284.x 18540879

[B69] Jarzebowski P, Hay YA, Grewe BF, Paulsen O (2022) Different encoding of reward location in dorsal and intermediate hippocampus. Curr Biol 32:834–841.e5. 10.1016/j.cub.2021.12.024 35016008

[B70] Jendryka M, Palchaudhuri M, Ursu D, van der Veen B, Liss B, Kätzel D, Nissen W, Pekcec A (2019) Pharmacokinetic and pharmacodynamic actions of clozapine-N-oxide, clozapine, and compound 21 in DREADD-based chemogenetics in mice. Sci Rep 9:1–14. 10.1038/s41598-019-41088-2 30872749 PMC6418145

[B71] Jin SW, Lee I (2021) Differential encoding of place value between the dorsal and intermediate hippocampus. Curr Biol 31:3053–3072.e5. 10.1016/j.cub.2021.04.073 34048706

[B72] Jinno S, Aika Y, Fukuda T, Kosaka T (1999) Quantitative analysis of neuronal nitric oxide synthase-immunoreactive neurons in the mouse hippocampus with optical disector. J Comp Neurol 412:398–412. 10.1002/(SICI)1096-9861(19990802)410:3<398::AID-CNE4>3.0.CO;2-9 10404408

[B73] Kawashima H, Izaki Y, Grace AA, Takita M (2006) Cooperativity between hippocampal-prefrontal short-term plasticity through associative long-term potentiation. Brain Res 1109:37–44. 10.1016/j.brainres.2006.06.034 16859647

[B74] Kelley AE, Domesick VB (1982) The distribution of the projection from the hippocampal formation to the nucleus accumbens in the rat: an anterograde and retrograde-horseradish peroxidase study. Neuroscience 7:2321–2335. 10.1016/0306-4522(82)90198-1 6817161

[B75] Kentner AC, Khoury A, Lima Queiroz E, MacRae M (2016) Environmental enrichment rescues the effects of early life inflammation on markers of synaptic transmission and plasticity. Brain Behav Immun 57:151–160. 10.1016/j.bbi.2016.03.013 27002704

[B76] Kheirbek MA, Drew LJ, Burghardt NS, Costantini DO, Tannenholz L, Ahmari SE, Zeng H, Fenton AA, Henl R (2013) Differential control of learning and anxiety along the dorsoventral axis of the dentate gyrus. Neuron 77:955–968. 10.1016/j.neuron.2012.12.038 23473324 PMC3595120

[B77] Kim J, Delcasso S, Lee I (2011) Neural correlates of object-in-place learning in hippocampus and prefrontal cortex. J Neurosci 31:16991–17006. 10.1523/JNEUROSCI.2859-11.2011 22114269 PMC3241739

[B79] Langston RF, Wood ER (2010) Associative recognition and the hippocampus: differential effects of hippocampal lesions on object-place, object-context and object-place-context memory. Hippocampus 20:1139–1153. 10.1002/hipo.20714 19847786

[B80] Langston R, Ainge J, Couey J, Canto C, Bjerknes T, Witter M, Moser E, Moser M (2010a) Development of the spatial representation system in the rat. Science 328:1576–1581. 10.1126/science.1188210 20558721

[B78] Langston RF, Stevenson CH, Wilson CL, Saunders I, Wood ER (2010b) The role of hippocampal subregions in memory for stimulus associations. Behav Brain Res 215:275–291. 10.1016/j.bbr.2010.07.006 20633579

[B82] Lee I, Park SB (2013) Perirhinal cortical inactivation impairs object-in-place memory and disrupts task-dependent firing in hippocampal CA1, but not in CA3. Front Neural Circuits 7:1–10. 10.3389/fncir.2013.00134 23966912 PMC3743073

[B81] Lee I, Hunsaker MR, Kesner RP (2005) The role of hippocampal subregions in detecting spatial novelty. Behav Neurosci 119:145–153. 10.1037/0735-7044.119.1.145 15727520

[B83] Leger M, Quiedeville A, Bouet V, Haelewyn B, Boulouard M, Schumann-Bard P, Freret T (2013) Object recognition test in mice. Nat Protoc 8:2531–2537. 10.1038/nprot.2013.155 24263092

[B84] Lesburguères E, Tsokas P, Sacktor T, Fenton A (2017) The object context-place-location paradigm for testing spatial memory in mice. Bio Protoc 7:1–12. 10.21769/BioProtoc.2231 PMC5697778 29170753

[B85] Lin X, Id MA, Blanton C, Avila B, Id TCH, Nitz DA, Id XX (2021) Noncanonical projections to the hippocampal CA3 regulate spatial learning and memory by modulating the feedforward hippocampal trisynaptic pathway. PLoS Biol 19:1–28. 10.1371/journal.pbio.3001127 PMC8741299 34928938

[B86] Liu P, Bilkey DK (2001) The effect of excitotoxic lesions centered on the hippocampus or perirhinal cortex in object recognition and spatial memory tasks. Behav Neurosci 115:94–111. 10.1037/0735-7044.115.1.94 11256456

[B87] Ma YY, Tian BP, Wilson FAW (2003) Dissociation of egocentric and allocentric spatial processing in prefrontal cortex. Neuroreport 14:1737–1741. 10.1097/00001756-200309150-00016 14512848

[B88] Malik R, Dougherty KA, Parikh K, Byrne C, Johnston D (2016) Mapping the electrophysiological and morphological properties of CA1 pyramidal neurons along the longitudinal hippocampal axis. Hippocampus 361:341–361. 10.1002/hipo.22526 PMC4760884 26333017

[B89] McKenna JT, Vertes RP (2004) Afferent projections to nucleus reuniens of the thalamus. J Comp Neurol 480:115–142. 10.1002/cne.20342 15514932

[B90] Milior G, Amalia M, Castro D, Sciarria LP, Garofalo S, Branchi I, Ragozzino D, Limatola C, Maggi L (2016) Electrophysiological properties of CA1 pyramidal neurons along the longitudinal axis of the mouse hippocampus. Nat Publ Gr 6:1–9. 10.1038/srep38242 PMC5138623 27922053

[B91] Mitchnick KA, Creighton S, O’Hara M, Kalisch BE, Winters BD (2015) Differential contributions of de novo and maintenance DNA methyltransferases to object memory processing in the rat hippocampus and perirhinal cortex - a double dissociation. Eur J Neurosci 41:773–786. 10.1111/ejn.12819 25639476

[B92] Moser MB, Moser EI (1998) Distributed encoding and retrieval of spatial memory in the hippocampus. J Neurosci 18:7535–7542. 10.1523/JNEUROSCI.18-18-07535.1998 9736671 PMC6793256

[B93] Mumby DG (2001) Perspectives on object-recognition memory following hippocampal damage: lessons from studies in rats. Behav Brain Res 127:159–181. 10.1016/S0166-4328(01)00367-9 11718890

[B94] Mumby DG, Gaskin S, Glenn MJ, Schramek TE, Lehmann H (2002) Hippocampal damage and exploratory preferences in rats: memory for objects, places, and contexts. Learn Mem 9:49–57. 10.1101/lm.41302 11992015 PMC155935

[B95] Nadel L (1968) Dorsal and ventral hippocampal lesions and behavior. Physiol Behav 3:891–900. 10.1016/0031-9384(68)90174-1

[B96] Neugebauer NM, Miyauchi M, Sato T, Tadano J, Akal H, Ardehali H, Meltzer HY (2018) Hippocampal GABA A antagonism reverses the novel object recognition deficit in sub-chronic phencyclidine-treated rats. Behav Brain Res 342:11–18. 10.1016/j.bbr.2017.12.033 29289597

[B97] Nieto-Escámez FA, Sánchez-Santed F, De Bruin JPC (2002) Cholinergic receptor blockade in prefrontal cortex and lesions of the nucleus basalis: implications for allocentric and egocentric spatial memory in rats. Behav Brain Res 134:93–112. 10.1016/S0166-4328(01)00458-2 12191796

[B98] Place R, Lykken C, Beer Z, Suh J, McHugh TJ, Tonegawa S, Eichenbaum H, Sauvage MM (2012) NMDA signaling in CA1 mediates selectively the spatial component of episodic memory. Learn Mem 19:164–169. 10.1101/lm.025254.111 22419815 PMC3312619

[B100] Poucet B (1989) Object exploration, habituation, and response to a spatial change in rats following septal or medial frontal cortical damage. Behav Neurosci 103:1009–1016. 10.1037/0735-7044.103.5.1009 2803548

[B99] Poucet B, Chapuis N, Durup M, Thinus-Blanc C (1986) A study of exploratory behavior as an index of spatial knowledge in hamsters. Anim Learn Behav 14:93–100. 10.3758/BF03200043

[B101] Ricceri L, Colozza C, Calamandrei G (2000) Ontogeny of spatial discrimination in mice : a longitudinal analysis in the modified open-field with objects. Dev Psychobiol 37:109–118. 10.1002/1098-2302(200009)37:2<109::AID-DEV6>3.0.CO;2-D 10954836

[B102] Roullet P, Sargolini F, Oliverio A, Mele A (2001) NMDA and AMPA antagonist infusions into the ventral striatum impair different steps of spatial information processing in a nonassociative task in mice. J Neurosci 21:2143–2149. 10.1523/JNEUROSCI.21-06-02143.2001 11245698 PMC6762623

[B103] Sabec MH, Wonnacott S, Warburton EC, Bashir ZI (2018) Nicotinic acetylcholine receptors control encoding and retrieval of associative recognition memory through plasticity in the medial prefrontal cortex. Cell Rep 22:3409–3415. 10.1016/j.celrep.2018.03.016 29590611 PMC5896173

[B104] Savalli G, Bashir ZI, Warburton EC (2015) Regionally selective requirement for D1/D5 dopaminergic neurotransmission in the medial prefrontal cortex in object-in-place associative recognition memory. Learn Mem 22:69–73. 10.1101/lm.036921.114 25593292 PMC4341361

[B105] Scott H, Smith A, Barker G, Uney J, Warburton E (2017) Contrasting roles for DNA methyltransferases and histone deacetylases in single-item and associative recognition memory. Neuroepigenetics 9:1–9. 10.1016/j.nepig.2017.02.001 28367410 PMC5364272

[B106] Sobin C, Flores-Montoya MG, Alvarez JM (2017) Early chronic low-level Pb exposure alters global exploratory behaviors but does not impair spatial and object memory retrieval in an object-in-place task in pre-adolescent C57BL/6J mice. Neurotoxicol Teratol 61:104–114. 10.1016/j.ntt.2017.01.002 28089843

[B107] Squire LR (1992) Memory and the hippocampus: a synthesis from findings with rats, monkeys, and humans. Psychol Rev 99:195–231. 10.1037/0033-295X.99.2.195 1594723

[B108] Squire LR (2004) Memory systems of the brain: a brief history and current perspective. Neurobiol Learn Mem 82:171–177. 10.1016/j.nlm.2004.06.005 15464402

[B109] Squire LR, Zola-Morgan S (1991) The medial temporal lobe memory system. Science 253:1380–1386. 10.1126/science.1896849 1896849

[B110] Strange BA, Witter MP, Lein ES, Moser EI (2014) Functional organization of the hippocampal longitudinal axis. Nat Rev Neurosci 15:655–669. 10.1038/nrn3785 25234264

[B111] Takita M, Izaki Y, Kuramochi M, Yokoi H, Ohtomi M (2010) Synaptic plasticity dynamics in the hippocampal-prefrontal pathway in vivo. Neuroreport 21:68–72. 10.1097/WNR.0b013e3283344949 19996810

[B112] Takita M, Fujiwara SE, Izaki Y (2013) Functional structure of the intermediate and ventral hippocampo-prefrontal pathway in the prefrontal convergent system. J Physiol Paris 107:441–447. 10.1016/j.jphysparis.2013.05.002 23719128

[B113] Taylor CJL, et al. (2025) Too little and too much : balanced hippocampal, but not medial prefrontal, neural activity is required for intact novel object recognition in rats. J Neurosci 45:1–12. 10.1523/JNEUROSCI.1141-25.2025 PMC12696617 41188028

[B114] Thompson CL, et al. (2008) Genomic anatomy of the hippocampus. Neuron 60:1010–1021. 10.1016/j.neuron.2008.12.008 19109908

[B115] Warburton EC, Brown MW (2015) Neural circuitry for rat recognition memory. Behav Brain Res 285:131–139. 10.1016/j.bbr.2014.09.050 25315129 PMC4383363

[B116] Wood SJ, Proffitt T, Mahony K, Smith DJ, Buchanan J-A, Brewer W, Stuart GW, Velakoulis D, McGorry P, Pantelis C (2002) Visuospatial memory and learning in first-episode schizophreniform psychosis and established schizophrenia : a functional correlate of hippocampal pathology ? Psychol Med 32:429–438. 10.1017/S0033291702005275 11989988

[B117] Wout MVT, Aleman A, Kessels RPC, Kahn RS (2006) Object-location memory in schizophrenia: interference of symbolic threatening content. Cogn Neuropsychiatry 11:272–284. 10.1080/13546800500214041 17354072

