# Supplementary figures and images for: Ventral and Intermediate Hippocampus Are Required for Object-in-Place Recognition Memory in Mice
Source: eNeuro. 2026 Jun 16;13(6):ENEURO.0105-26.2026. doi: 10.1523/ENEURO.0105-26.2026 (PMC13271815; doi:10.1523/ENEURO.0105-26.2026)

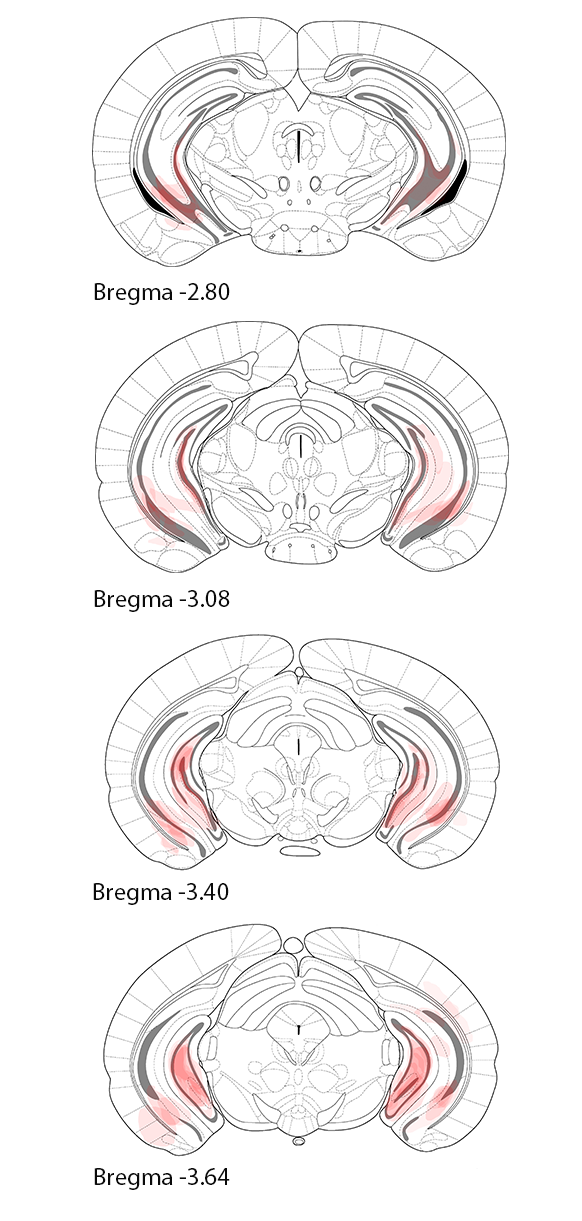

Supplement: Figure 1-1 — Viral expression maps of vCA1 manipulations. Diagrams depicting overlaid viral spread for all vCA1 hM4D infused animals. Download Figure 1-1, TIF file. [file eneuro-13-ENEURO.0105-26.2026-s001.tif]

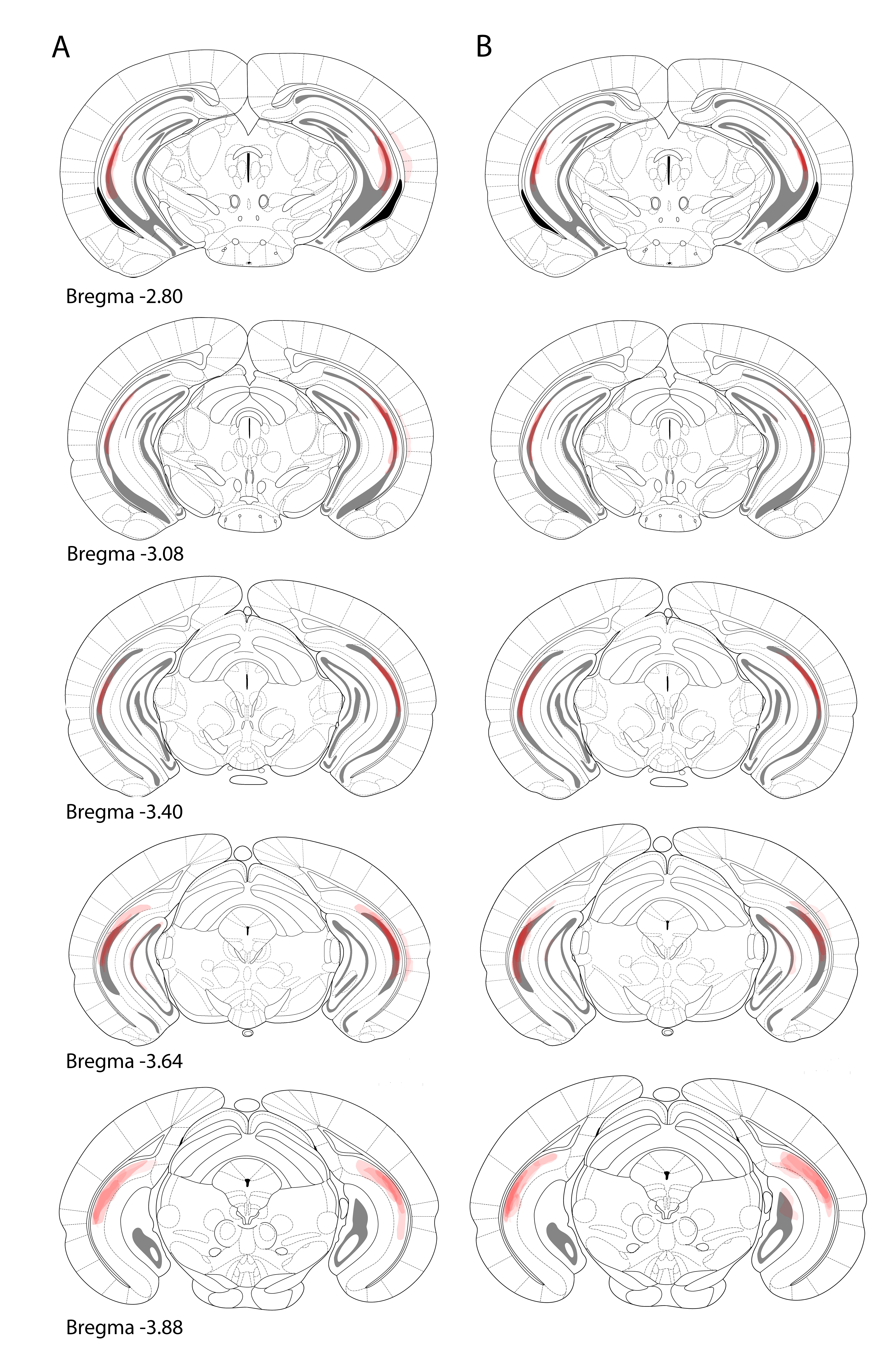

Supplement: Figure 2-1 — Viral expression maps of iCA1 manipulations. Diagrams depicting overlaid viral spread for all iCA1 hM4D mice that underwent the OiP task in the allocentric (A) or egocentric (B) configurations. Download Figure 2-1, TIF file. [file eneuro-13-ENEURO.0105-26.2026-s002.tif]

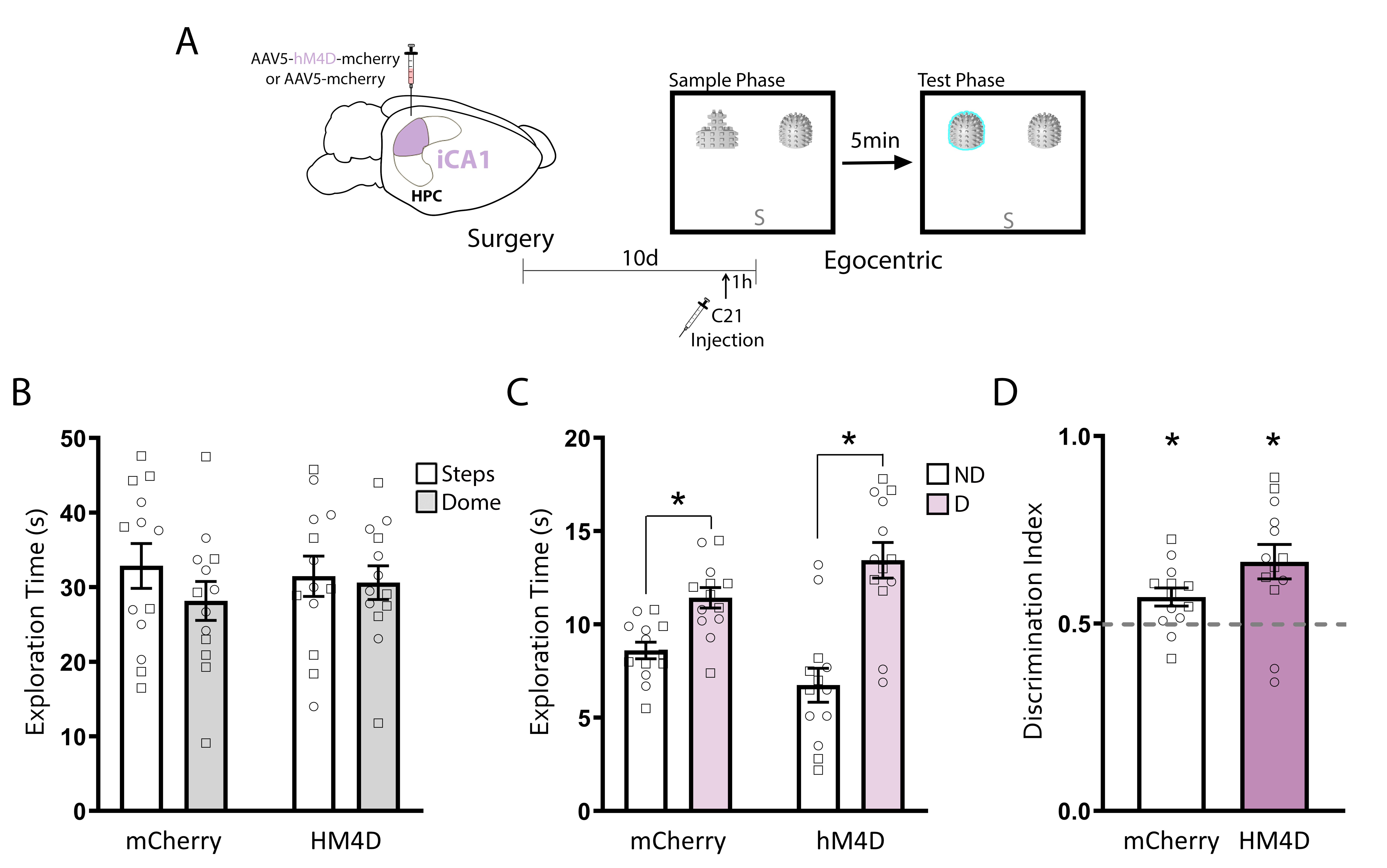

Supplement: Figure 2-2 — iCA1 activity is not necessary for egocentric two-object OiP memory. iCA1 manipulation during egocentric two-object OiP. A. Schematic of the task design for the egocentric version of the two-object OiP task. Animals underwent identical procedures as in Figure 1A, except that during the test phase they were placed in the chamber facing the same wall as the sample phase (depicted with an S in the scheme). B. mCherry and hM4D iCA1 mice showed no object preference during the sample phase (two-way ANOVA, no treatment x object type interaction: F(1,24)­ = 1.116, p = 0.3013; main effect of object: F(1,24) = 2.311, p = 0.1514 or of treatment: F(1,24) = 0.02565, p = 0.8741). C. During the test phase, hM4D iCA1 egocentric mice spent more time exploring the displaced (D) compared to the non-displaced (ND) objects. D. Calculation of a discrimination index for each animal showed that both mCherry and hM4D iCA1 egocentric animals displayed a preference for the displaced object vs chance levels. mCherry: n = 13 (6 females, 7 males); hM4D: n = 13 (6 females, 7 males). Individual datapoints from female mice are depicted as circles and male mice as squares for transparency, but no sex differences were found. *p < 0.05. Download Figure 2-2, TIF file. [file eneuro-13-ENEURO.0105-26.2026-s003.tif]

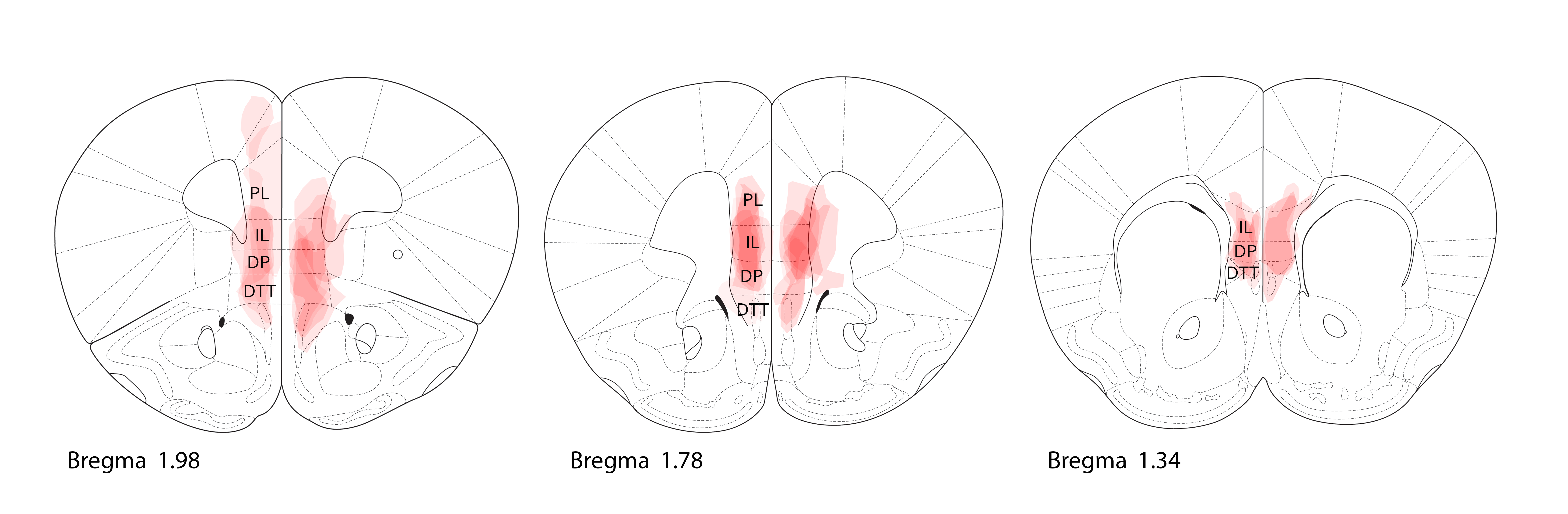

Supplement: Figure 3-1 — Viral expression maps of mPFC manipulations. Diagrams depicting overlaid viral spread for all mPFC hM4D infused animals. PL = Prelimbic cortex, IL = Infralimbic cortex, DP = Dorsal peduncular Cortex, DTT = Dorsal tenia tecta. Download Figure 3-1, TIF file. [file eneuro-13-ENEURO.0105-26.2026-s004.tif]
